# Free Cholesterol‐Induced Liver Injury in Non‐Alcoholic Fatty Liver Disease: Mechanisms and a Therapeutic Intervention Using Dihydrotanshinone I

**DOI:** 10.1002/advs.202406191

**Published:** 2024-11-19

**Authors:** Jia‐Wen Shou, Juncai Ma, Xuchu Wang, Xiao‐Xiao Li, Shu‐Cheng Chen, Byung‐Ho Kang, Pang‐Chui Shaw

**Affiliations:** ^1^ Li Dak Sum Yip Yio Chin R&D Centre for Chinese Medicine The Chinese University of Hong Kong Hong Kong 852852 China; ^2^ Centre for Cell and Developmental Biology State Key Laboratory for Agrobiotechnology School of Life Sciences The Chinese University of Hong Kong Hong Kong 852852 China; ^3^ Department of Laboratory Medicine the Second Affiliated Hospital of Zhejiang University Hangzhou 310000 China; ^4^ Research Center for Chinese Medicine Innovation The Hong Kong Polytechnic University Hong Kong 852852 China; ^5^ School of Nursing The Hong Kong Polytechnic University Hong Kong 852852 China; ^6^ School of Life Sciences The Chinese University of Hong Kong Hong Kong 852852 China; ^7^ State Key Laboratory of Research on Bioactivities and Clinical Applications of Medicinal Plants and Institute of Chinese Medicine The Chinese University of Hong Kong Hong Kong 852852 China

**Keywords:** NAFLD, Free cholesterol, ROS, Lysosome, Dihydrotanshinone I, PPARα

## Abstract

Build‐up of free cholesterol (FC) substantially contributes to the development and severity of non‐alcoholic fatty liver disease (NAFLD). Here, we investigate the specific mechanism by which FC induces liver injury in NAFLD and propose a novel therapeutic approach using dihydrotanshinone I (DhT). Rather than cholesterol ester (CE), we observed elevated levels of total cholesterol, FC, and alanine transaminase (ALT) in NAFLD patients and high‐cholesterol diet‐induced NAFLD mice compared to those in healthy controls. The FC level demonstrated a positive correlation with the ALT level in both patients and mice. Mechanistic studies revealed that FC elevated reactive oxygen species level, impaired the function of lysosomes, and disrupted lipophagy process, consequently inducing cell apoptosis. We then found that DhT protected mice on an HCD diet, independent of gut microbiota. DhT functioned as a potent ligand for peroxisome proliferator‐activated receptor α (PPARα), stimulating its transcriptional function and enhancing catalase expression to lower reactive oxygen species (ROS) level. Notably, the protective effect of DhT was nullified in mice with hepatic PPARα knockdown. Thus, these findings are the first to report the detrimental role of FC in NAFLD, which could lead to the development of new treatment strategies for NAFLD by leveraging the therapeutic potential of DhT and PPARα pathway.

## Graphical abstract

This study underscores the detrimental role of free cholesterol in non‐alcoholic fatty liver disease (NAFLD), demonstrating its ability to trigger a surge in reactive oxygen species (ROS) that leads to hepatocyte death. We also introduce dihydrotanshinone I (DhT), a small molecule that mitigates free cholesterol‐induced toxicity by activating peroxisome proliferator‐activated receptor α, thereby reducing ROS levels. This finding points to a promising new treatment strategy for NAFLD.

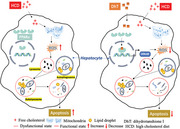

## 1 Introduction

Non‐alcoholic fatty liver disease (NAFLD) encompasses a wide range of conditions resulting from the accumulation of fat in the liver, including simple non‐alcoholic fatty liver (NAFL) and non‐alcoholic steatohepatitis (NASH), which may progress to fibrosis, cirrhosis, or even hepatocellular carcinoma.^[^ ^1^ ^]^ Currently, NAFLD is one of the major causes of morbidity and a healthcare burden worldwide, with a global prevalence higher than 30%.^[^ ^2^ ^]^ The current treatment mainly lies in lifestyle modifications, physical activity and pharmacological interventions; however, the benefits resulted from those treatment options are far from satisfaction due to low compliance and poor real‐world efficacies.^[^ ^3^ ^]^ The research and development of novel medication against NAFLD is slow‐moving, with the first Food and Drug Administration‐approved drug (Rezdiffra) only announced in March 2024, which is used to treat adults with noncirrhotic NASH with moderate to advanced fibrosis and to be used along with diet and exercise.^[^ ^4^ ^]^ Therefore, much more effort should be taken to develop novel therapies.

NAFLD has traditionally been characterized by increased hepatic triglyceride accumulation.^[^ ^5^ ^]^ Recent studies have however revealed disturbance of cholesterol hemostasis as another crucial factor in the pathogenesis of NAFLD.^[^ ^6^ ^]^ Lipidomic analysis of NAFLD patients has demonstrated an increased accumulation of free cholesterol (FC), without a similar increase in cholesterol esters (CE), compared to healthy controls.^[^ ^7^ ^]^ FC has been well documented to be cytotoxic in several aspects. It is directly associated with inflammation and fibrosis in NAFLD,^[^ ^8^ ^]^ potentially through mechanisms that involve increasing Toll‐like receptor 4 signaling.^[^ ^9^ ^]^ FC also caused hepatocyte death by activating c‐Jun N‐Terminal Protein Kinase 1.^[^ ^10^ ^]^ Additionally, FC accumulation is observed in the development of liver cancer associated with fatty liver.^[^ ^11^ ^]^ Notably, recent studies have shown that hepatocyte death is one of the key triggers of NAFLD severity, leading to the subsequent development of inflammation, fibrosis, cirrhosis, and even hepatocellular carcinoma.^[^ ^12^ ^]^ However, the detailed mechanism by which cell death or injury responds to cholesterol accumulation (both FC and CE) in NAFLD remains yet to be fully understood.

Given that cholesterol is closely involved in the pathology of NAFLD, cholesterol‐lowering therapy has been advocated and adopted in the management of this disease.^[^ ^11^, ^13^ ^]^ Statins no doubt have been the first choice.^[^ ^14^ ^]^ However, physicians are cautious about prescribing statins to patients with NAFLD, due to the concerns that statins might induce asymptomatic elevation in serum alanine transaminase (ALT),^[^ ^15^ ^]^ trigger autoimmune hepatitis,^[^ ^16^ ^]^ and rhabdomyolysis.^[^ ^17^ ^]^ Thus, statins are underused in NAFLD patients.^[^ ^15^, ^18^ ^]^ There is still an urgent need for seeking novel and effective pharmaceutical agents.

*Salvia miltiorrhiza* Bge. (known as Danshen in Chinese) has been clinically shown to be effective in treating NAFLD, whether used alone or as part of an herbal formula.^[^ ^19^ ^]^ *Salvia miltiorrhiza Bge*. manages NAFLD involving the regulation of several key factors, including c‐Jun N‐terminal kinases, sterol regulatory element‐binding protein‐1c, carbohydrate response element binding proteins, peroxisome proliferator‐activated receptors (PPARs), and cytochrome P450.^[^ ^20^ ^]^ One of the major tanshinones isolated from *Salvia miltiorrhiza* Bge, dihydrotanshinoneI(DhT), has been shown to have cardiovascular protective, anti‐inflammatory, anti‐oxidant, and hepaprotective actions.^[^ ^21^ ^]^ However, the therapeutic effect and mechanism of DhT in treating NAFLD are still unknown.

In this study, we observed elevated levels of TC and FC in both NAFLD patients and high‐cholesterol diet (HCD)‐induced NAFLD mice compared to the healthy control, with a positive correlation with ALT levels. Rather than CE, FC was found to be detrimental to hepatocytes, as it increased the level of reactive oxygen species (ROS), impaired lysosome functions, and ultimately inhibited lipophagy, leading to cell apoptosis. Treatment of mice on an HCD with DhT alleviated FC‐induced cell apoptosis by activating PPAR𝛼 and promoting catalase expression, subsequently down‐regulating the FC‐triggered burst of ROS.

## 2 Results

### 2.1 The level of FC is significantly upregulated in NAFLD and positively correlated with the ALT level

**Figure 1:**
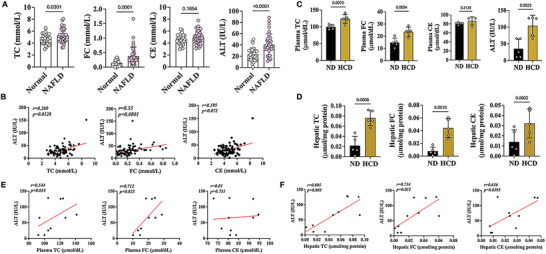
FC was positively correlated with hepatic injury in both NAFLD patients and mice. (A) Plasma levels of TC, FC, CE and ALT in human. (B) Correlation analysis between ALT value and TC, FC or CE levels in human. (C) Plasma levels of TC, FC, CE and ALT in mice. (D) Hepatic levels of TC, FC, CE and ALT in mice. (E‐F) Correlation analysis between ALT value and TC, FC or CE levels in mice plasma (E) and liver (F).

NAFLD is accompanied by dyslipidemia and hepatic injury in clinic. To investigate the relationship between cholesterol (CHO) levels and hepatic injury, we collected plasma from 36 NAFLD patients and 30 normal healthy controls to detect the levels of TC, FC, CE, and ALT, and to analyze their correlations. Figure S1A‐C show the basic information of all individuals. Our findings revealed increased levels of TC, FC, and ALT in clinic plasma samples (**Figure** **1**A). Spearman correlation analysis showed a significant positive correlation between FC and ALT (Figure 1B), indicating the potential toxic role of FC in NAFLD.

Then, we established an NAFLD mouse model using a HCD.^[^ ^22^ ^]^ The successful model establishment was evidenced by increased body weight gain, higher liver index and NAFLD scores, and elevated levels of lipids and CHO compared to mice fed a normal diet (Figure 1C, D; S1D‐I). The NAFLD mouse model produced similar outcomes to the human patients, demonstrating a positive correlation between FC and ALT in both the liver tissue and plasma (Figure 1E, F).

Given that CHO can be transformed into oxysterols to maintain CHO hemostasis in the liver,^[^ ^23^ ^]^ gas chromatography–mass spectrometry was used to detect the major metabolites. Our findings revealed that an HCD notably increased the hepatic CHO levels, but there was no statistically significant increase in the levels of the five oxysterols (Figure S2A). Additionally, we did not observe changes in CE levels (Figure 1C, D) or alterations in cholesterol esterase expression as a result of the HCD (Figure S2B).

These findings underscore the positive association between FC and liver injury in NAFLD, observed in both humans and mice, suggesting the potential toxic effects of FC.

### 2.2 FC triggered cell apoptosis

**Figure 2:**
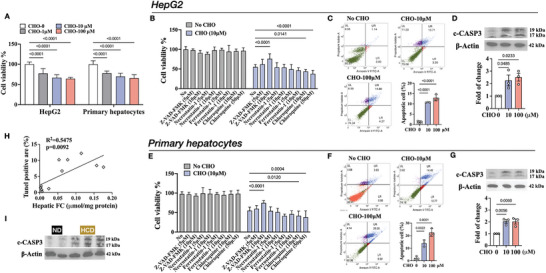
FC triggered cell apoptosis. (A) Cell viability with CHO treatment. (B‐D) Cell viability with CHO and different cell death inhibitors treatment (B), flow analysis of apoptotic cells (C), and immunoblotting of c‐CASP3 (D) in HepG2 cells. (E‐G) Cell viability with CHO and different cell death inhibitors treatment (B), flow analysis of apoptotic cells (C), and immunoblotting of c‐CASP3 (D) in primary hepatocytes. (H‐I) Correlation analysis between TUNEL positive area and hepatic FC level (H) and immunoblotting of hepatic c‐CASP3 (I) in mice fed with ND or HCD.

To find out what was the impact of FC on the liver, we treated HepG2 cell line and mouse primary hepatocytes with CHO, and different cell death inhibitors were applied. In **Figure** **2**A, CHO followed a dose‐dependent manner to lower the cell viability, which showed higher toxicity in comparison with the same doses of CE (Figure S2C). At non‐toxic doses, Z‐VAD‐FMK, a pan‐caspase inhibitor of apoptosis, was observed to protect both HepG2 cells and primary hepatocytes from CHO‐induced toxicity (Figure 2B, E). Flow cytometry‐based apoptosis analysis revealed that CHO promoted cell apoptosis (Figure 2C, F) and resulted in an increase in the level of cleaved‐CASPASE3 (c‐CASP3) (Figure 2D, G). Furthermore, filipin staining of FC in mouse liver sections demonstrated that HCD administration led to a significant FC accumulation (Figure S2D), which is consistent with the results obtained using a biochemical kit (Figure 1D). TUNEL staining of liver sections confirmed that HCD promoted cell apoptosis (Figure S2E). Spearman correlation analysis indicated a strongly positive correlation between the FC level and the TUNEL positive area in mouse livers (Figure 2H). Immunoblotting of c‐CASP3 further supported the notion that FC increased cell apoptosis in NAFLD mouse livers (Figure 2I, S3D). Therefore, we concluded that FC could trigger cell apoptosis in NAFLD mouse livers.

### 2.3 FC induced lysosome dysfunction

In addition to the apoptosis‐promoting effect of CHO, we also observed that the presence of chloroquine, an autophagy inhibitor, could exacerbate CHO toxicity on cell viability (Figure 2B, E). Considering the complex interactions between apoptosis and autophagy,^[^ ^24^ ^]^ we sought to investigate whether autophagic flux played a role in the apoptotic death induced by CHO.

Initially, we employed both an agonist (Rapamycin) and antagonists (Bafilomycin A1 and chloroquine) to co‐treat cells with CHO. The results indicated that both the agonist and the antagonists exacerbated the CHO‐induced toxicity, as evidenced by the lower cell viability compared to cells treated with CHO only (Figure S2F, G). Subsequently, we delved into investigating how CHO impacted the autophagic flux.

**Figure 3:**
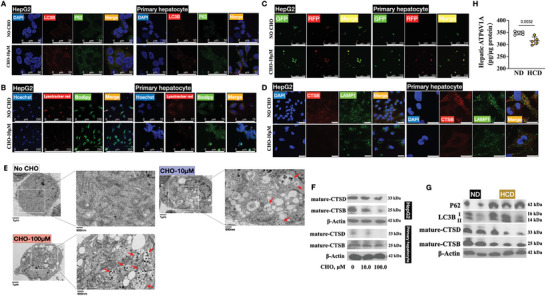
FC induced lysosome dysfunctions. (A) Representative co‐immunofluorescence staining of DAPI, LC3B and P62 in HepG2 cells and primary hepatocytes without and with CHO treatment, scale bar: 50 µm. (B) Representative co‐staining of Hoechst 33342, lysotracker red and bodipy 505/515 in HepG2 cells and primary hepatocytes without and with CHO treatment, scale bar: 250 µm. (C) Representative fluorescence detection of HepG2 cells and primary culture transfected with a plasmid encoding GFP‐RFP‐LC3 without and with CHO treatment, scale bar: 250 µm. (D) Representative co‐immunofluorescence staining of DAPI, CTSB and LAMP1 in HepG2 cells and primary hepatocytes without and with CHO treatment, scale bar: 50 µm. (E) Transmission electron microscopy detection of autolysosomes (red arrow) in HepG2 cells without and with CHO treatment. (F) Immunoblotting of CTSB and CTSD in HepG2 cells and primary hepatocytes without and with CHO treatment. (G) Immunoblotting of hepatic P62, LC3B, CTSB and CTSD in mice fed with ND or HCD. (H) Hepatic ATP6V1A content detected using an Elisa kit in mice fed with ND or HCD.

Subsequently, co‐immunostaining of microtubule‐associated proteins 1A/1B light chain 3B (LC3B) and ubiquitin‐binding protein p62 (P62), two autophagic markers, revealed that CHO treatment not only increased the puncta and expression of LC3B and P62 but also promoted an increase in the co‐localization of these two markers (**Figure** **3**A; S1A, BS3A, B). As autophagy in NAFLD is closely related to lipid degradation, commonly referred to as lipophagy, we proceeded to investigate the co‐localization of lipids and the lysosome. With CHO treatment, bodipy staining demonstrated elevated lipid accumulation in both HepG2 cells and primary hepatocytes, while lysosome puncta following lysotracker red staining showed reduced intensity, resulting in a decreased level of overlapped puncta (Figure 3B).

Next, the cells were transfected with a tandem mRFP‐GFP‐LC3 construct to investigate autolysosome formation. The assessment was based on the principle that the low pH inside the lysosome quenches the GFP fluorescent signal, while the mRFP fluorescence is more stable in an acidic environment.^[^ ^25^ ^]^ Therefore, normal autolysosome maturation would result in more RFP‐only puncta. However, if lysosome function is impaired, most of the puncta would display both red and green signals, appearing yellow. As illustrated in Figure 3C, cells challenged with CHO exhibited yellow signals. Additionally, we performed immunostaining of two proteins, cathepsin B (CTSB) and lysosomal‐associated membrane protein 1 (LAMP1), to confirm the impaired state of the lysosome, as CSTB is processed via proteases in lysosomes into a mature form, and LAMP1 represents a lysosomal membrane protein. As expected, CHO treatment resulted in the impairment of the lysosome, as evidenced by the significant downregulation of overlapped puncta of CTSB and LAMP1 (Figure 3D). Transmission electron microscopy was utilized to observe the lysosome and autolysosome, and the data demonstrated that autolysosomes accumulated after CHO treatment (Figure 3E), indicating the dysfunction of lysosomes.

Then, we examined the expression of LC3B, P62, CTSB, and cathepsin D (CTSD) in both in vitro and in vivo samples. The results showed that CHO treatment increased P62 levels and the ratio of LC3BII to LC3BI (Figure S3A, B, D; 3G). The reduced levels of mature CTSB and CTSD following CHO treatment indicated lysosome impairment (Figure 3F, G; S3C, D). Additionally, we detected the expression of a key enzyme, V‐type proton ATPase catalytic subunit A (ATP6V1A), in liver homogenate as it is crucial for maintaining the acidic environment in lysosomes.^[^ ^26^ ^]^ Mice on an HCD showed decreased hepatic ATP6V1A activity, compared to the mice on an ND (Figure 3H).

In summary, we could conclude that FC induced lysosome dysfunction.

### 2.4 FC triggered ROS burst to impair lysosome functions

**Figure 4:**
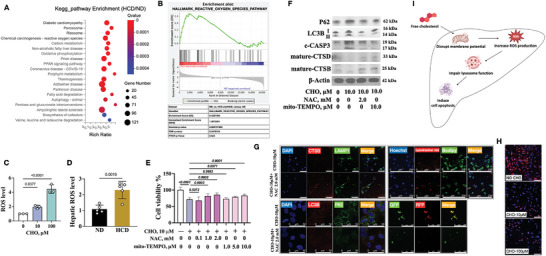
Oxidative stress involved in FC‐induced toxicity. (A) KEGG pathway enrichment of differential expressed genes from HCD mice versus ND mice. (B) GSEA analysis of reactive oxygen species pathway in HCD and ND mice. (C‐D) ROS levels in HepG2 cells challenged with CHO (C) and ND or HCD mouse liver (D). (E) HepG2 cell viability with CHO, NAC and mito‐TEMPO treatment for 24 hours. (F) Immunoblotting of P62, LC3B, c‐CASP3, CTSB and CTSD in HepG2 cells with CHO, NAC or mito‐TEMPO treatment. (G) HepG2 cells post‐treatment of CHO and NAC with co‐immunofluorescence staining of DAPI, CTSB and LAMP1 (upper left, scale bar: 50 µm); co‐staining of Hoechst 33342, lysotracker red and bodipy 505/515 (upper right, scale bar: 250 µm); co‐staining of LC3B, P62 and DAPI (lower left, scale bar: 50 µm); and fluorescence detection after transfected with a plasmid encoding GFP‐RFP‐LC3 (lower right, scale bar: 250 µm). (H) Mitochondrial membrane potential of HepG2 cells with CHO treatment (scale bar: 100 µm).

To find out how FC impaired lysosome, hepatic transcriptome study was performed. Differential gene expression between mice fed on a ND and those on a HCD was analyzed (Figure S4A) and subjected to KEGG pathway enrichment analysis. **Figure** **4**A illustrates the most significant pathways, and the involvement of pathways related to NAFLD confirmed the successful establishment of the model. Furthermore, gene set enrichment analysis (GSEA) revealed the presence of autophagy, apoptosis, and inflammatory response pathways in HCD mice compared to ND mice (Figure S4B‐D), consistent with our immunostaining and immunoblotting data (Figure 2, 3).

Given that oxidative stress has been well documented in lysosome impairment,^[^ ^27^ ^]^ we paid close attention in the oxidation related pathways. GSEA demonstrated that HCD was positively related to the ROS pathway (Figure 4B), and CHO treatment upregulated the ROS level both in vitro and in vivo (Figure 4C, D). Scavenging ROS using N‐acetyl‐l‐cysteine (NAC) or Mito‐TEMPO exhibited protective effects on HepG2 cells challenged by CHO (Figure 4E). Furthermore, NAC treatment reduced CHO‐induced lysosome impairment, as evidenced by decreased expression and co‐localization of autophagic markers (P62 and LC3B), increased maturation of CTSB and CTSD and their overlap with LAMP1, as well as a reduction in merged puncta between lipids and lysosomes (Figure 4F, G; S4E). As mitochondria are a major source of ROS and mitochondrial membrane potential is directly associated with ROS burst,^[^ ^28^ ^]^ we subsequently measured the mitochondrial membrane potential using tetramethyl rhodamine ethyl ester (TMRE). In Figure 4H, CHO treatment demonstrated a dose‐dependent manner in disrupting membrane potential.

Therefore, we concluded that FC disrupted the membrane potential of mitochondria to induce ROS burst, subsequently impairing lysosome functions (Figure 4I).

### 2.5 DhT mitigated NAFLD in mice on an HCD, irrespective of gut microbiota

DhT, extracted from *Salvia miltiorrhiza* Bunge, has been extensively researched for its ability to alleviate oxidative stress ^[^ ^29^ ^]^. However, it was previously unclear whether DhT is effective in mitigating NAFLD by suppressing HCD‐induced oxidative damage.

**Figure 5:**
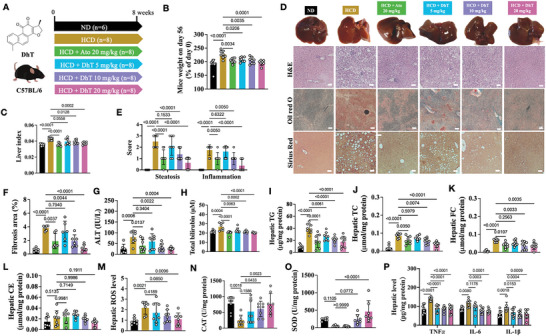
DhT mitigated NAFLD in HCD‐fed mice. (A) Structure of DhT and experimental groups in this study. (B‐C) Mouse weight (B) and liver index (C) on day 56. (D) H&E, oil red O and Sirius red staining of mouse liver sections. (E‐F) NAFLD score (E) and fibrosis area (F) in mouse liver. (G‐H) Liver function indices, ALT (G) and total bilirubin (H). (I‐L) Hepatic levels of TG (I), TC (J), FC (K) and CE (L). (M‐O) Hepatic ROS (M) and antioxidases (N, Catalase; O, SOD) level. (P) Hepatic inflammatory markers level.

In this study, in addition to the HCD, mice received low (5 mg/kg), medium (10 mg/kg), and high (20 mg/kg) doses of DhT (dissolved in 0.5% CMC‐Na), with the vehicle serving as the negative control (**Figure** **5**A). Furthermore, atorvastatin (Ato, 20 mg/kg) was used as a positive control (Figure 5A). Following an eight‐week treatment period, it was observed that both DhT and Ato reduced mice weight gain (Figure 5B) and decreased the liver index (Figure 5C). Moreover, histological analysis revealed that DhT mitigated liver damage in mice challenged with HCD, as evidenced by fewer vacuoles, reduced cell infiltration, less lipid accumulation, and decreased collagen deposition compared to the mice administered with the vehicle (Figure 5D, E, F). Additionally, DhT downregulated levels of ALT and total bilirubin, further supporting the hepatic protective effect of DhT (Figure 5G, H). As expected, Ato treatment exhibited similar efficacy in this mouse model (Figure 5D, E, F, G, H).

Furthermore, lipid and CHO (including TC, FC and CE) quantifications demonstrated the beneficial regulatory actions of both DhT and Ato. There was a significant decline in TG, TC, and FC levels after DhT or Ato treatment, except for CE (Figure 5I, J, K, L). Additionally, ROS triggered by HCD was notably downregulated, accompanied by a significant increase in the expression of antioxidant proteins including catalase and superoxide dismutase (SOD) (Figure 5M, N, O). Moreover, the anti‐inflammatory effect of DhT was evidenced by a decrease in the levels of tumor necrosis factor alpha (TNFα), Interleukin 6 (IL‐6), and IL‐1β (Figure 5P).

As gut microbiota is actively involved in the pathological and therapeutic aspects of NAFLD,^[^ ^30^ ^]^ it is reasonable to hypothesize the possibility that DhT might follow a gut microbiota‐dependent manner to mitigate NAFLD. To explore this impact, we conducted an analysis of changes in gut microbiota by performing 16S rRNA sequencing of fecal samples from mice that were on a ND, an HFD, as well as two different doses of DhT (10mg/kg, DhT10; 20mg/kg, DhT20). No significant changes were observed in the Ace and Chao indices for alpha diversity following DhT administration (Figure S5A, B). Beta diversity was only evident between the mice fed with the ND and the HCD, and DhT did not lead to adjustments in the microbial community (Figure S5C). Further analysis of specific microbial taxa at the phylum and family levels yielded similar results, indicating that DhT was ineffective at reshaping the HCD‐disturbed gut microbiota (Figure S5D, E). However, it is worth noting that while the HCD decreased the gut microbiota health index, DhT was found to increase this index (Figure S5F, G).

To further explore the role of gut microbiota in the effectiveness of DhT on NAFLD mice, we employed a pseudo germ‐free model to determine whether DhT provided protection for NAFLD mice with depleted gut microbiota. Establishing a pseudo germ‐free state involved the administration of antibiotics every other day, which was confirmed by qPCR analysis of fecal 16S rRNA levels (Figure S6A, B). In this pseudo germ‐free state, DhT demonstrated beneficial effects for NAFLD mice, including reduced body and liver weight, decreased steatosis, inflammation, and fibrosis, preserved liver function, and enhanced antioxidant capacity (Figure S6C‐N). These findings suggest that gut microbiota is not essential for DhT to exert its protective effects on NAFLD.

### 2.6 DhT protected lysosome from FC‐induced damage

Although promoting CHO excretion may be a protective mechanism,^[^ ^23^ ^]^ DhT administration did not increase the fecal TC level (Figure S7A), suggesting a minimal role of DhT in cholesterol excretion. Therefore, the protective function of DhT on HCD‐fed mice might be attributed to interactions between DhT and FC.

**Figure 6:**
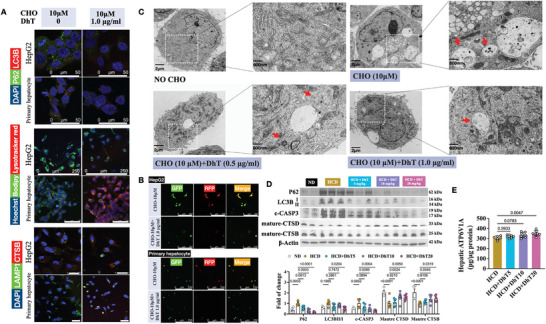
DhT reversed FC‐ induced lipophagy blockage. (A) Co‐staining of DAPI, P62 and LC3B (upper, scale bar: 50 µm); Hoechst 33342, lysotracker red and bodipy 505/515 (middle, scale bar: 250 µm); DAPI, CTSB and LAMP1 (lower, scale bar: 50 µm) in HepG2 cells and primary hepatocytes with CHO and DhT treatment. (B) Fluorescence detection after transfected with a plasmid encoding GFP‐RFP‐LC3 in HepG2 cells and primary hepatocytes with CHO and DhT treatment, scale bar: 250 µm. (C) Transmission electron microscopy detection of autolysosomes (red arrow) in HepG2 cells with CHO and DhT treatment. (D) Immunoblotting and quantification of hepatic P62, LC3B, CTSB and CTSD in HCD mice with DhT treatment. (E) Hepatic ATP6V1A content detected using an Elisa kit in HCD mice with DhT treatment.

As shown in Figure S7B, DhT protected HepG2 cells and primary hepatocytes from CHO‐induced toxicity. We previously confirmed that CHO induced cell apoptosis through impairing the functions of lysosomes. Here, DhT has been shown to reduce the expression and co‐localization of P62 and LC3B (**Figure** **6**A; S7C, D). DhT treatment resulted in decreased lipid accumulation and reduced co‐localization of lipids with lysosomes. However, the overlap of CTSB and LAMP1 puncta was increased in both HepG2 cells and primary hepatocytes (Figure 6A; S7C, D).

In addition, the lipid accumulation and co‐localization between lipid and lysosome has been downregulated, whereas the overlapped puncta of CTSB and LAMP1 has been increased by DhT treatment in both HepG2 cells and primary hepatocytes (Figure 6A; S7C, D). Plus, yellow signals after merging the GFP and RFP channels decreased after DhT treatment, suggesting the normal acidic environment of lysosome (Figure 6B). Transmission electron microscopy analysis demonstrated that DhT reduced autolysosomes accumulation induced by CHO treatment (Figure 6C).

We next detected the level of autophagy markers (P62 and LC3B), apoptotic markers (c‐CASP3 and TUNEL staining), and lysosome function indicators (CTSB and CTSD) in NAFLD mouse liver samples. As expected, DhT reversed the detrimental effect of HCD following a dose‐dependent manner (Figure 6D, S7C‐E). The level of ATP6V1A, a key factor to maintain the acidic environment in lysosomes, was promoted after DhT administration (Figure 6E), implying the recovered lysosomal functions.

### 2.7 Peroxisome proliferator‐activated receptor alpha (PPAR𝛼) is involved in the effect of DhT

**Figure 7:**
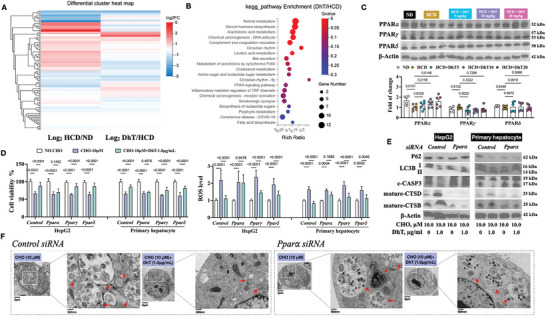
PPAR𝛼 is involved in the effect of DhT. (A) Differential expressed genes among ND, HCD and DhT mice. (B) KEGG pathway enrichment of differential expressed genes from HCD mice versus HCD+DhT mice. (C) Immunoblotting and quantification of PPARs in mouse liver. (D‐E) Cell viability (D left), ROS content (D right) and immunoblots of P62, LC3B, c‐CASP3, mature CTSB and mature CTSD (E) in siRNA‐transfected HepG2 and primary hepatocytes with CHO and DhT treatment. (F) Transmission electron microscopy detection of autolysosomes (red arrow) in siRNA‐transfected HepG2 cells with CHO and DhT treatment.

Transcriptome analysis in mouse liver was applied to find out by which mechanism DhT was able to exert protection on NAFLD mice. First, we have observed that the disturbed genes by HCD could be reversed by DhT treatment (**Figure** **7**A), which indicated the therapeutical efficacy of DhT on mice challenged by HCD. KEGG pathway analysis of differentially expressed genes identified from DhT‐treated mice versus HCD mice revealed the involvement of the PPAR signaling pathway (Figure 7B), aligning with the concept that PPARs are potential therapeutic targets for NAFLD.^[^ ^31^ ^]^ Next, the expression of three isoforms of PPARs, including PPAR𝛼, PPAR𝛾 and PPAR𝛿, have been detected both in vitro and in vivo. With DhT treatment, PPAR𝛼 was found to be promoted, while the expression of PPAR𝛾 and PPAR𝛿 was not altered significantly (Figure 7C, S8A).

Subsequently, siRNA‐mediated gene silencing was applied to elucidate the specific role of each PPAR isoform. The successful gene silencing was confirmed by the reduced expression at the translational level (Figure SB). We assessed cell viability and ROS levels post‐siRNA transfection followed by CHO and DhT treatment. The protective effect of DhT on CHO‐challenged cells (HepG2 and primary hepatocytes) was diminished upon PPAR𝛼 silencing, with similar cell viability and ROS levels compared to cells treated with CHO only (Figure 7D).

Next, autophagy markers (P62 and LC3B), apoptotic marker (c‐CASP3), and lysosome function indicators (CTSB and CTSD) were detected in cells with PPARs silencing. Similar to previous data, DhT was able to regulate those markers in cells with control siRNA transfection, and knockdown of PPAR𝛼, rather than PPAR𝛾 and PPAR𝛿, failed to exhibit the protective action of DhT (Figure 7E, S8C). Additionally, autolysosomes displayed using transmission electron microscopy analysis gave another piece of evidence to elucidate the importance of PPAR𝛼 (Figure 7F).

Therefore, we could conclude that PPAR𝛼 is key to the effect of DhT on NAFLD.

### 2.8 DhT activated PPAR𝛼 to promote catalase expression

**Figure 8:**
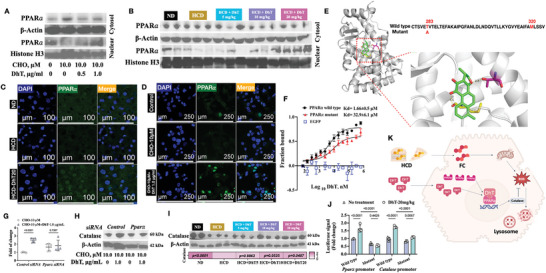
DhT bound and activated PPAR𝛼 to promote catalase expression. (A‐B) PPAR𝛼 expression and localization in cytosol and nuclear fractions in HepG2 cells (A, C) and mouse liver (B, D) with CHO and DhT treatment. C: scale bar: 250 µm; D: scale bar: 100 µm. (E) Docking simulation of PPAR𝛼 LBD (PDB: 3vi8) and DhT. (F) Binding affinity between PPAR𝛼 LBD wild type and mutant using microscale thermophoresis. (G‐H) Catalase expression in siRNA‐transfected HepG2 with CHO and DhT treatment at the transcriptional (G) and translational (H) levels. (I) Level of catalase in mouse liver with HCD and DhT treatment. (J) Promoter activities of Ppar𝛼 and catalase in HepG2 cells with CHO and DhT treatment. (K) Graphical summary illustrating the protective mechanism of DhT.

PPAR𝛼 is a ligand‐activated transcription factor that governs the metabolic homeostasis. Upon activation, PPAR𝛼 translocates into the nucleus to exert its transcriptional regulation on target genes.^[^ ^32^ ^]^ The distribution of PPAR𝛼 was examined using immunoblotting and immunostaining in HepG2 cells and mouse liver sections. Both techniques revealed that DhT promoted the nuclear distribution of PPAR𝛼 (**Figure** **8**A‐D). In addition to the increased nuclear distribution, we observed the expression of the confirmed downstream target genes of PPAR𝛼, including fatty acid transport protein 1 (*Fatp1*), carnitine palmitoyl transferase 1 alpha (*Cpt1𝛼*) and acyl‐CoA oxidase 1 (*Acox1*), could be elevated by DhT (Figure S8D). Thus, DhT has been confirmed to activate PPAR𝛼.

Subsequently, after conducting a simulation of the protein‐ligand binding interaction using Autodock 4.2, we observed that Threonine 283 (T283) and Methionine 320 (M320) formed a binding pocket, anchoring DhT within the ligand binding domain (LBD) of PPAR𝛼 with a binding energy of ‐9.38 kcal/mol (Figure 8E). We next expressed, purified, and concentrated recombinant PPAR𝛼 LBD fused with an EGFP tag for the subsequent binding interaction analysis (Figure S9). The binding affinity (Kd) between DhT and PPAR𝛼 LBD was measured using microscale thermophoresis. It was observed that EGFP did not interact with DhT, making the Kd calculation unfeasible (Figure 8F). Notably, the Kd between DhT and PPAR𝛼 LBD was determined to be 1.66 ± 0.5 μM (Figure 8F), indicating a possible interaction. Given DhT interacted with the main chain of M320 and the side chain of T283 in PPAR𝛼 we subsequently introduced a mutation where threonine was changed to alanine (T283A) (Figure 8E), and the Kd was re‐evaluated. The mutated protein exhibited a lower affinity, resulting in an increased Kd of 32.9 ± 6.1 μM (Figure 8F).

Since DhT treatment led to the decrease in hepatic ROS content in NAFLD mice, we screened for potential antioxidants in wild‐type cells and PPAR𝛼‐silenced cells. Data showed that DhT elevated the levels of catalase, SOD1, SOD2, thioredoxin 1 (TRX1) in cells with CHO treatment (Figure 8G, H;  S8E); whereas PPAR𝛼 knockdown abolished the action of DhT to promote catalase expression at the transcriptional and translational levels (Figure 8G, H;  S8C). Additionally, the effect of DhT to promote catalase expression was observed in NAFLD mice following a dose‐dependent manner (Figure 8I).

We further investigated the transcriptional regulation of PPAR𝛼 and catalase expression mediated by DhT‐activated PPARα using a luciferase assay. It is known that PPAR𝛼 can regulate its own expression by binding to its promoter upon activation by an agonist.^[^ ^33^ ^]^ In our study, DhT‐activated PPAR𝛼 increased the activity of the *PPAR𝛼* promoter (Figure 8J), leading to higher PPAR𝛼 expression. Additionally, we observed an increase in the luciferase signal of *catalase* promoter in the presence of DhT‐activated PPAR𝛼 (Figure 8J). However, this effect was not observed when PPAR𝛼 was mutated (Figure 8J).

In summary, we concluded that DhT activated PPAR𝛼 to promote catalase expression, which could down‐regulate the ROS level induced by HCD (Figure 8K).

### 2.9 Adeno‐associated virus (AAV)‐mediated PPAR𝛼 knockdown reduced the protective effect of DhT on NAFLD mice

**Figure 9:**
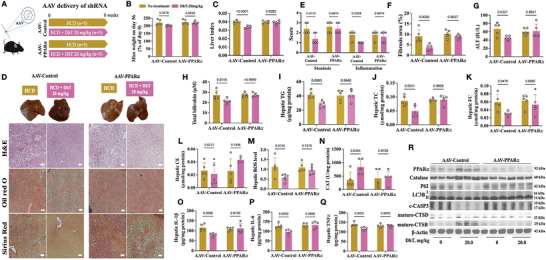
Hepatic PPAR𝛼 knockdown reduced the effect of DhT. (A) Experimental grouping in this study. (B–C) Mouse weight (B) and liver index (C) on day 56. (D) H&E, oil red O and Sirius red staining of mouse liver sections. (E–F) NAFLD score (E) and fibrosis area (F) in mouse liver. (G–H) Liver function indices, ALT (G) and total bilirubin (H). (I–L) Hepatic levels of TG (I), TC (J), FC (K) and CE (L). (M–N) Hepatic ROS (M) and catalase (N) level; (O–P) Hepatic inflammatory markers level. (R) Expressions of PPAR𝛼, catalase, P62, LC3B, c‐CASP3, mature CTSB and mature CTSD in AAV mouse liver.

To further explore the importance of PPAR𝛼, we applied AAV‐mediated shRNA delivery to reduce the hepatic PPAR𝛼 level, followed by the NAFLD model establishment and DhT treatment (**Figure** **9**A). shRNA efficiencies were verified in murine cells lines and shRNA‐1 was selected for the in vivo experiment (Figure S10).

In mice receiving control shRNA (AAV‐Control), DhT was beneficial to reduce body weight gain (Figure 9B), liver index (Figure 9C), NAFLD score (Figure 9D, E), fibrosis area (Figure 9F) and level of TG (Figure 9I), TC (Figure 9J), FC (Figure 9K) and inflammatory markers (Figure 9O, P, Q). In addition, DhT maintained the liver function (Figure 9G, H) and increased antioxidant capacity (Figure 9M, N). Levels of PPAR𝛼, catalase, autophagy markers (P62 and LC3B), apoptotic marker (c‐CASP3), and lysosome function indicators (CTSB and CTSD) showed the similar trends (Figure 9R) after DhT treatment (Figure 6, 7 and 8I).

The successful hepatic PPAR𝛼 knockdown was confirmed by the reduced PPAR𝛼 expression detected by the immunoblotting (Figure 9R). Further, the action of DhT to promote PPAR𝛼 was prohibited (Figure 9R). As expected, the effect of DhT to ameliorate NAFLD was weakened as shown in Figure 9B‐R. Therefore, PPAR𝛼 is pivotal to the protective effect of DhT on NAFLD.

## 3 Discussion

CHO is an important lipid molecule for animal cells, maintaining the barrier function between the environment and cells, modulating fluidity of biological membranes and forming lipid rafts that concentrate signaling molecules.^[^ ^23^ ^]^ Under the physiological condition, cellular CHO levels are determined by the interplay between de novo biosynthesis, uptake, export and storage to maintain the CHO hemostasis.^[^ ^34^ ^]^ The liver is a key organ involved in the regulation of CHO hemostasis, and hepatic CHO is found in the form of FC and CE, both of which are constituents of circulating lipoproteins.^[^ ^34^ ^]^ Besides the de novo synthesis, absorption from the diet is another major source of hepatic CHO. Thus, HCD containing 0.25 to 2% cholesterol has been widely used to establish NAFLD models.^[^ ^35^ ^]^

Here, despite the unchanged CE content, higher FC level was observed in NAFLD patients and HCD‐induced NAFLD mice, in consistent with the previous reports.^[^ ^7^, ^36^ ^]^ Though FC has been one of the lipotoxic chemicals, we are the first to report the positive correlation between the level of FC with that of ALT. As metabolizing CHO into oxysterols could detoxify the CHO‐induced toxicity, we thereby detected the levels of major oxysterols and found that, with HCD administration, only CHO content was promoted. Therefore, there might be a potential link between FC and NAFLD pathology.

Despite the previously well‐documented mechanism of saturated fatty acid‐mediated lipotoxicity in the liver, recent case‐control studies and follow‐up research have shown that hypercholesterolemia or high consumption of CHO, rather than the intake of fat or saturated fatty acid, was associated with a higher risk of NAFLD.^[^ ^37^ ^]^ FC, rather than CE, has been widely confirmed to play a significant role in this context.^[^ ^6^, ^7^, ^36^, ^38^ ^]^ The toxicity of FC has been addressed in both parenchymal cells (hepatocytes) and non‐parenchymal cells (Kupffer cells and stellate cells) in the liver, and the injury of each cell type would aggravate FC‐induced damage to the other cells. The accumulation of FC, rather than triglycerides or fatty acids, increases the sensitivity of hepatocytes to inflammatory cytokines‐mediated steatohepatitis via decreasing the level of mitochondrial glutathione.^[^ ^39^ ^]^ CHO crystals, accumulated and precipitated in the damaged hepatocytes, contribute to triggering an inflammatory response by interacting with NLRP3 inflammasomes in Kupffer cells.^[^ ^40^ ^]^ Activation of hepatic stellate cells has been involved in FC accumulation and has been reported to regulate Toll‐like receptor 4 signaling^[9a]^ or increase the sensitivity transforming growth factor beta (TGFβ)‐induced activation in a vicious cycle,^[9b]^ leading to fibrosis in NASH. Additionally, as an endpoint outcome of FC‐induced toxicity, cell death has been described in this NAFLD. Being the predominant mode of cell death, the presence of apoptotic cell death has been observed in clinical samples as evident by the higher level of caspases or TUNEL positive signals,^[^ ^41^ ^]^ which is possible due to subcellular dysfunctions including endoplasmic reticulum stress, mitochondrial dysfunction and lysosomal dysfunction.^[38b]^ Besides, necroptosis, pyroptosis and autophagy‐related death have been reported yet less understood than apoptosis in NAFLD.^[^ ^42^ ^]^ Thus, there lacks a detailed explanation to decipher how hepatocytes response to FC challenge, as well as a thorough mechanism by which FC kills or damages the normal cellular functions.

As a defense mechanism in NAFLD, autophagy degrades accumulated lipids inside hepatocytes, a process termed lipophagy.^[^ ^43^ ^]^ However, lipophagy is functionally impaired in NAFLD patients, leading to lipid overaccumulation.^[^ ^44^ ^]^ In this study, we discovered that FC initially promotes autophagy induction but subsequently blocks the later autophagic degradation process. This finding is consistent with previous reports that hepatic autophagy is enhanced in the early stages of NAFLD but is blocked at later stages.^[^ ^45^ ^]^ Our study provides a molecular mechanism for this phenomenon: FC accumulation increases ROS production, damaging lysosomal function and thereby blocking the later stages of lipophagy. This insight offers a novel perspective on why lipophagy fails in NAFLD.

Given that FC is more toxic than CE in HepG2 cells and primary hepatocytes, and that HCD administration increases TUNEL‐positive cells in liver sections, we investigated whether cell death was related to lipophagy blockage. By using various inhibitors targeting different cell death pathways (apoptosis, necroptosis, ferroptosis, and autophagy), we found that FC induces caspase‐dependent apoptosis. Additionally, in the presence of an autophagy agonist (rapamycin) or antagonists (chloroquine and bafilomycin A1), FC further enhances cell death. A myriad of evidence demonstrates crosstalk between autophagy (or lipophagy) and apoptosis.^[^ ^46^ ^]^ The interconnection between autophagy and apoptosis is complex, often occurring in sequence, with autophagy preceding apoptosis. In most cases, autophagy can remove toxic or obsolete proteins and organelles, thereby reducing the tendency of cells to undergo apoptosis.^[^ ^47^ ^]^ As lysosomes are responsible for autophagy‐related degradation, their dysfunction leads to autophagy blockage and debris accumulation, ultimately inducing apoptosis.^[^ ^48^ ^]^ The widely accepted mechanism explaining lysosome dysfunction‐related apoptosis involves mitochondrial participation. Exposed to stimuli such as growth factor deprivation, hypoxia, DNA damage, and oxidative stress, mitochondria directly trigger caspase activation or induce ROS bursts that rupture lysosomes, releasing cysteine proteases to augment apoptotic factors.^[48b]^ We propose that FC increases ROS levels, leading to lysosome dysfunction. Therefore, FC toxicity is attributed to ROS‐induced lysosomal damage. Our observation provides the first explanation for why FC accumulation is toxic in the liver.

CHO‐lowering medications, including but not limited to atorvastatin, are beneficial to ameliorate NAFLD from this study as well as others’ research.^[^ ^14^, ^15^ ^]^ Patients receiving statins treatment not only exhibited lower lipid and CHO levels but also showed decreased c‐CASP3 content and increased catalase levels (Figure S1J, K). Moreover, we have introduced DhT as a new potential candidate for not only lowering CHO levels but also attenuating FC‐induced toxicity, which, to our best of knowledge, it is the first to report this efficacy. PPARs are ligand‐activated transcription factors of nuclear hormone receptor superfamily comprising of the following three subtypes including PPAR𝛼, PPAR𝛾 and PPARδ. Each PPAR consists of an LBD and a DNA binding domain. After interacting with ligands, PPARs are translocated to the nucleus, where they activate their transcriptional function by binding to specific DNA regions of target genes known as peroxisome proliferator hormone response elements.^[^ ^31^ ^]^ PPARs have emerged as integrators of energy homeostasis and metabolic function, and their pivotal roles in disease management have well been documented in metabolic disorders, cancers and liver diseases.^[^ ^49^ ^]^ Due to crucial metabolic regulatory roles and excellent draggability, PPARs have been widely discussed clinically in the treatment of metabolic disorders, including NAFLD. For example, PPAR𝛼 agonists, such as Fenofibrate and Gemfibrozil, have demonstrated reliable improvement of liver function and lipid profiles. However, they have only exhibited minimal effects on histopathology in NAFLD.^[^ ^50^ ^]^ Pioglitazone, a PPAR𝛾 selective agonist, has been confirmed effective in treating patients with NAFLD and type 2 diabetes. However, the efficacy to treat NAFLD patients without type 2 diabetes is limited.^[^ ^51^ ^]^ The research progress of PPAR𝛿 agonists has not been as promising as others. For example, Seladelpar's clinical trial has been discontinued due to atypical histological findings.^[^ ^52^ ^]^ What is more, PPAR dual/pan agonists, including Lanifibranor and Saroglitazar, have been developed for treating NAFLD.^[^ ^52^ ^]^ Apart from the beneficial effects of these agonists, adverse events such as impaired kidney function, rhabdomyolysis, weight gain, and cardiovascular dysfunctions have also been reported.^[^ ^52^, ^53^ ^]^ Therefore, addressing the resolution of NAFLD remains a challenging task, requiring further concerted efforts.

Our data confirm that DhT is a promising candidate for treating NAFLD. This study is the first to report that DhT is a novel and potent ligand for PPAR𝛼. As a ligand‐inducible transcription factor, DhT binds to and activates PPAR𝛼, thereby triggering its transcriptional regulatory actions. We observed that DhT‐activated PPAR𝛼 increased the activity of the *Ppar𝛼* promoter, indicating a self‐regulatory effect, which is a typical characteristic of PPARs.^[^ ^33^, ^49^, ^50^, ^51^, ^52^, ^53^, ^54^ ^]^^[33,49b‐d,54]^ Given that oxidative stress is a key contributor to the detrimental effects induced by FC accumulation, we investigated whether the beneficial effects of PPAR𝛼 in NAFLD were related to its antioxidant action. Knockdown of PPAR𝛼 abolished the antioxidant effect of DhT. Next, we screened for antioxidant genes regulated by DhT‐activated PPAR𝛼 and identified catalase as a potential downstream target. Similar to *Ppar𝛼*, catalase expression was upregulated following DhT treatment, which was achieved through increased recruitment of the *catalase* promoter to DhT‐activated PPAR𝛼.

Although the PPAR𝛼‐catalase pathway has been reported in the treatment of alcoholic liver disease,^[^ ^55^ ^]^ hepatic fibrosis,^[^ ^56^ ^]^ and skin aging,^[^ ^57^ ^]^ this study is the first to elucidate its role in mitigating FC‐induced oxidative stress in NAFLD through DhT treatment. Furthermore, while the antioxidant effects of DhT have been previously reported in stroke^[^ ^58^ ^]^ and atherosclerosis,^[^ ^59^ ^]^ this is the first study to demonstrate that antioxidant action of DhT is effective in treating NAFLD, highlighting its potential as a therapeutic agent. To more comprehensively evaluate the anti‐NAFLD effects of DhT, we suggest that additional NAFLD models beyond the HCD‐induced model should be employed, which will be a focus of our future research efforts.

In summary, our work delineate that FC is toxic in the pathology of NAFLD, owing to the detrimental effect of FC to induce lysosome dysfunctions to provoke hepatocyte apoptosis. In addition, we propose a novel PPAR𝛼 agonist, DhT, to treat NAFLD, through a mechanism by which DhT activates and binds to PPAR𝛼 agonist to down‐regulate FC‐induced ROS burst, therefore showing protective efficacy.

## 4 Experimental Section

### Human samples

This study was approved by the Ethics Committee of the Second Affiliated Hospital, Zhejiang University School of Medicine (Approval No. 2019–420). The study was non‐interventional and did not involve additional treatment, operations, or examinations on the subjects, and did not pose any adverse reaction or risk. Thirty normal healthy controls, thirty‐six NAFLD patients without treatment (clinically confirmed by physicians), and thirty NAFLD patients after statin treatment were enrolled from the outpatient records after obtaining written consent. Plasma samples were collected to determine lipid, cholesterol (CHO) and ALT levels.

### Animal study

The animal studies described in this research were approved and carried out in accordance with the guidelines of the University Animal Experimentation Ethics Committee at The Chinese University of Hong Kong (21‐210‐MIS). Male four‐week‐old C57BL/6J mice, provided by Laboratory Animal Services Centre at The Chinese University of Hong Kong, were used to establish the NAFLD model.

### Animal study—High‐cholesterol diet (HCD) induced NAFLD mouse model

HCD was prepared by adding 1% CHO (sc‐202539A, Santa Cruz, Dallas, TX, USA) to an HFD consisting of 60% fat.^[49b]^ The NAFLD model was induced by administering HCD for eight weeks, while the normal chow diet (ND) was used as the control. Plasma and liver samples were collected after the eight‐week administration.

### Animal study—Drug treatment on HCD‐induced NAFLD mice

Dihydrotanshinone I (DhT, SD8290, Solarbio) was administered to mice by gavage every other day during the HCD‐fed period at doses of 5, 10, and 20 mg/kg, dissolved in 0.5% CMC‐Na solution (HY‐Y1889, MedChemExpress, Monmouth Junction, NJ, USA). Atorvastatin (Ato, A6800, Solarbio) was used as a positive control. The vehicle (0.5% CMC‐Na solution) was the negative control for both ND‐ or HCD‐fed mice. The grouping information is presented in Figure 5A.

### Animal study—Pseudo‐germ‐free mouse model establishment and DhT treatment

A pseudo‐germ‐free model was created by administering a mixture of antibiotics, including cefadroxil (50 mg/kg; IC0160, Solarbio), terramycin (50 mg/kg; IO0130, Solarbio), and erythromycin (50 mg/kg; E8100, Solarbio) every other day.^[^ ^60^ ^]^ Fecal 16S rDNA levels were assessed using qPCR analysis every two weeks to confirm the pseudo‐germ‐free state. The NAFLD model was established as above at 4.2.2 during the antibiotics administration period, and DhT (20 mg/kg) was orally administered to these mice every other day. The grouping information is presented in Figure S6A.

### Animal study—Adeno‐associated virus (AAV)‐mediated hepatic PPAR𝛼 knockdown and DhT treatment

The AAV containing PPAR𝛼 shRNA was prepared following our previous work.^[^ ^49c^ ^]^ In vivo knockdown of PPAR𝛼 was achieved by tail‐vein injection of AAV (1 × 10^12^ gc/mouse in 50 μl of normal saline), followed by HCD administration to establish the NAFLD state. Additionally, DhT (20 mg/kg) was administered orally to these mice every other day. The grouping information is presented in Figure 9A.

### Primary hepatocytes isolation

Primary hepatocytes were isolated from mice (three mice at a time) following a published protocol.^[^ ^61^ ^]^ After anesthesia, the liver was perfused, hepatocytes were dissociated using collagenase, separated from other cells, and cultured for subsequent experiments.

### Cell culture

HepG2 cells (ATCC, Manassas, VA, USA) and primary hepatocytes were cultured in Dulbecco's Modified Eagle's Medium (DMEM, 11965092, Thermo Fisher Scientific, Waltham, MA, USA) supplemented with 10% fetal bovine serum (A5256701, Thermo Fisher Scientific) and 1% penicillin/streptomycin (15140122, Thermo Fisher Scientific).

### MTT assay

3‐(4,5‐dimethylthiazol‐2‐yl)‐2,5‐diphenyltetrazolium (MTT) assay (M6494, Thermo Fisher Scientific) was used to assess cell viability. Cells were seeded onto 96‐well plates and treated for 24 hours with the following compounds: CHO (sc‐202539A, Santa Cruz, Dallas, TX, USA; 1, 10, and 100 μM), cholesterol esters (cholesterol palmitate, sc‐214704, Santa Cruz: 10 and 100 μM; cholesterol stearate, sc‐214708, Santa Cruz 10 and 100 μM), DhT (0.1, 0.5, and 1 µg/mL), N‐acetyl‐l‐cysteine (NAC, A7250, Sigma‐Aldrich, Saint Louis, MO, USA: 0.1, 0.2, and 2.0 mM), Mito‐TEMPO (SML0737, Sigma‐Aldrich: 1, 5, and 10 μM), chloroquine (14194, Cayman chemical, Ann Arbor, MI, USA: 10 and 50 μM), Z‐VAD‐FMK (C1202, Beyotime, Beijing, China: 5 and 10 μM), necrostatin‐1 (SC4359, Beyotime, Beijing, China: 5 and 10 μM), ferrostatin‐1 (17729, Cayman chemical: 5 and 10 μM), bafilomycin A1 (S1413, Selleckchem, Houston, TX, USA: 25 nM), or rapamycin (sc‐3504, Santa Cruz: 500 nM).

### Flow cytometer analysis (ROS and Apoptosis)

Cellular and hepatic levels of reactive oxygen species (ROS) were measured using a Total ROS Assay kit (88‐5930‐74, Thermo Fisher Scientific). The probe from the kit was added to the cells (after CHO or/and DhT treatment) or liver homogenate and incubated for 30 minutes, followed by flow cytometric analysis.

Cell apoptosis was assessed using an Annexin V‐FITC cell apoptosis detection kit (C1062S, Beyotime). Flow cytometry was employed to detect apoptosis in both HepG2 cells and primary hepatocytes challenged with CHO or/and DhT.

### Biochemical assays

Levels of lipids, total cholesterol (TC), free cholesterol (FC), alanine transaminase (ALT), triglycerides (TG), high density lipoprotein cholesterol (HDL‐c), low density lipoprotein cholesterol (LDL‐c), cleaved CASPASE3 (c‐CASP3), catalase, V‐type proton ATPase catalytic subunit A (ATP6V1A), superoxide dismutase (SOD), tumor necrosis factor alpha (TNFα), Interleukin 6 (IL‐6) and IL‐1β were detected using the following kits: TC content assay kit (BS1985, Solarbio); FC content assay kit (BS1895, Solarbio); ALT activity assay Kit (ab105134, Abcam, Cambridge, UK), Stanbio™ Triglyceride Liquid Reagent (2100430, Fisher Scientific, Pittsburgh, PA, USA), HDL‐c (BC5325, Solarbio), LDL‐c (BC5335, Solarbio), human cleaved caspase‐3 (Asp175) ELISA kit (ab220655, Abcam), catalase activity assay kit (ab83464, Abcam; BC0205, Solarbio), mouse ATP6V1A ELISA kit (RK13939, ABclonal, Wuhan, China), SOD activity kit (BC5165, Solarbio), mouse TNFα (SEKM‐0034, Solarbio), mouse IL‐6 (SEKM‐0007, Solarbio) and mouse IL‐1β (SEKM‐0002, Solarbio).

### siRNA transfection

Transfection of control siRNA (sc‐37007, Santa Cruz), Pparα siRNA (human: sc‐36307; mouse: sc‐36308; Santa Cruz), Pparγ siRNA (human: sc‐29455; mouse: sc‐29456; Santa Cruz) or Pparδ siRNA (human: sc‐36305; mouse: sc‐36306) was carried out using Lipofectamine 2000 (11668027, Thermo Fisher Scientific) as recommended by the manufacturer. Then, cells with siRNA transfection were subject to CHO and DhT treatment as above.

### Transmission Electron Microscopy

HepG2 cells treated with CHO and DhT were collected and washed three times with PBS. The cells were then fixed with a mixture of 2% formaldehyde and 2.5% glutaraldehyde in 0.1 M Sodium Cacodylate buffer (pH 7.4) at 4 °C. After three times of washing with 0.1 M Sodium Cacodylate buffer, the cells were post‐fixed with 1% OsO_4_ for 1 hour at room temperature. Then cells were undergoing dehydration using a graded acetone series (10%‐100%). Subsequently, samples were embedded in Embed‐812 resin (14120, Electron Microscopy Sciences, Hatfield, PA). Thin sections (100 nm thick) from the sample blocks were examined using a Hitachi 7400 TEM (Hitachi‐High Technologies, Tokyo, Japan) operated at 80 kV.

### Immunoblot

Total protein from cells or tissues was extracted using RIPA lysis and extraction buffer (89900, Thermo Fisher Scientific) and quantified with the Pierce™ BCA Protein Assay Kit (23227, Thermo Fisher Scientific). Nuclear and cytosol fractions were isolated using NE‐PER™ Nuclear and Cytoplasmic Extraction Reagents (78833, Thermo Fisher Scientific). The following antibodies were used for immunoblotting: PPARα (sc‐398394, Santa Cruz), PPARγ (2435T, Cell Signaling Technology, Danvers, MA, USA), PPARδ (GTX113250, GeneTex, Irvine, CA, USA), cleaved Caspase‐3 (c‐CASP3, 9664T, Cell Signaling Technology), LC3B (3868T, Cell Signaling Technology), SOAT1 (sc‐137013, Santa Cruz), SOAT2 (sc‐69837, Santa Cruz), cathepsin D (CTSD, sc‐377299, Santa Cruz), cathepsin B (CTSB, sc‐365558, Santa Cruz), LC3B (3868T, Cell Signaling Technology), P62 (sc‐48402, Santa Cruz), catalase (A11220, ABclonal), Histone H3 (9715S, Cell Signaling Technology), and β‐Actin (sc‐47778, Santa Cruz).

### AAV preparation

AAV‐ short hairpin RNA (shRNA)‐ctrl was a gift from Hongjun Song (Addgene plasmid # 85741; http://n2t.net/addgene:85741; RRID: Addgene 85741). pAAV2/8 and pAdDeltaF6 plasmids were gifts from James M. Wilson (Addgene plasmid # 112864; http://n2t.net/addgene:112864; RRID: Addgene 112864; Addgene plasmid # 112867; http://n2t.net/addgene:112867; RRID: Addgene_112867). The shRNA sequence targeting PPAR𝛼 (Accession: NM_001113418.1) was designed using an online tool‐ https://biosettia.com/support/shrna‐designer/ (Table S1). An AAV‐shRNA‐ctrl (AAV‐Control) plasmid was constructed to incorporate the PPAR𝛼 shRNA (AAV‐ PPAR𝛼). AAV packaging was performed according to our previous publication^[49c]^ AAV titer was calculated using qPCR (Table S1)

### RNA extraction, real‐time PCR analysis and RNA Sequencing

RNA from cells and tissues was extracted from cells and tissues with a RNAeasy™ Animal Long RNA Isolation Kit with Spin Column (R0027, Beyotime) and quantified using NanoDrop™ One/OneC Microvolume UV‐Vis Spectrophotometer (Thermo Fisher Scientific). cDNA conversion from mRNA was conducted using a BeyoRT™ III First Strand cDNA Synthesis Kit (D7178M, Beyotime). mRNA levels were analyzed with Luna Universal qPCR Master Mix (M3003L, New England Biolabs, Ipswich, MA, USA). Primers are listed in Table S2.

Meanwhile, RNA samples were sent to BGI Tech Solutions Co., Ltd. (Hong Kong) for RNA sequencing. After assessing RNA integrity and quantification, the downstream sequencing process involved removing ribosomal RNA before library construction, followed by mRNA fragmentation, cDNA synthesis, and fragment enrichment. The libraries were then loaded onto the Illumina HiSeq4000 platform for sequencing, and the data were analyzed using the Standard Dr. Tom analysis. The transcriptome datasets of the current study are available in the Sequence Read Archive Database in National Center for Biotechnology Information with the accession number (PRJNA1118074).

### Histological examination and immunostaining

Buffered‐formalin‐fixed, paraffin‐embedded liver sections were stained with hematoxylin and eosin (H&E) or Sirius red to assess histopathological severity and fibrosis. Frozen liver sections were subject to Oil Red O staining to evaluate lipid accumulation.

If necessary, paraffin‐embedded and frozen liver sections underwent antigen retrieval using an Abcam heat‐induced epitope retrieval protocol. Then, liver sections or cells were stained with DAPI (62248, Thermo Fisher Scientific), Hoechst 33342 (62249, Thermo Fisher Scientific), Filipin (sc‐205323, Santa Cruz), TUNEL Assay Kit – HRP‐DAB (ab206386, Abcam), LC3B (3868T, Cell Signaling Technology), P62 (sc‐48402, Santa Cruz), Lysotracker Red (C1046, Beyotime), BODIPY™ 493/503 (D3922, Thermo Fisher Scientific), CTSB (sc‐365558, Santa Cruz), LAMP1 (sc‐17768, Santa Cruz), Mitochondrial Membrane Potential Assay Kit with TMRE (C2001S, Beyotime), PPARα (sc‐398394, Santa Cruz).

All the images of stained sections or cells were captured using Carl Zeiss PALM Inverted Microscope (Carl Zeiss, Oberkochen, Germany) and Leica TCS SP8 Confocal Microscope System (Leica Microsystems, Wetzlar, Germany).

### Fecal genomic DNA extraction and 16SrRNA sequencing

Feces were collected from mice following DhT treatment and promptly stored at −80 °C prior to analysis. Fecal genomic DNA extraction and 16S rRNA sequencing were conducted in accordance with our previous publication.^[49c]^

### Protein expression and purification

The coding sequence for the human PPAR𝛼‐ligand binding domain (LBD) (GenBank accession number NM_005036.6, containing amino acids 200–468) was cloned into the expression vector pET28a to produce a recombinant protein fused with a N‐terminal HIS6‐EGFP tag. Mutated PPAR𝛼 LBD was obtained PCR‐aided site directed mutation (T283A). *Escherichia coli* BL21(DE3) containing the constructed plasmids (wild‐type or mutated construct) was cultured in 4 L of luria broth medium at 37 °C until reaching an OD_600_ value of 0.4. The culture was then cooled to 10 °C and induced with 0.1 mM IPTG for 30 hours. Cells were harvested by centrifugation at 8000 rpm, resuspended in lysis buffer (20 mM Tris pH 8.0, 150 mM NaCl, 5 mM imidazole, 10% glycerol, and 10 mM β‐mercaptoethanol), and lysed with a JN‐Mini Low Temperature Ultra‐High Pressure Cell Disrupter (JNBio, China). The resulting supernatant was collected after centrifugation at 20,000 rpm for 60 minutes, and then loaded onto a 2 mL Ni2+ column. The column was washed with 50 mL of wash buffer 1 (20 mM Tris pH 8.0, 150 mM NaCl, 10 mM imidazole, 10% glycerol), 50 mL of wash buffer 2 (20 mM Tris pH 8.0, 200 mM NaCl, 15 mM imidazole, 10% glycerol), 50 mL of wash buffer 3 (20 mM Tris pH 8.0, 200 mM NaCl, 15 mM imidazole, 10% glycerol), and 50 mL of wash buffer 4 (20 mM Tris pH 8.0, 150 mM NaCl, 20 mM imidazole, 10% glycerol), followed by elution of bound protein with buffer 5 (20 mM Tris pH 8.0, 150 mM NaCl, 25–500 mM imidazole, 10% glycerol, 10 mM β‐mercaptoethanol). The eluted protein was concentrated with an Amicon® Stirred Cell (MERCK, Rahway, NJ, USA) and the buffer was changed to buffer 5 (20 mM Tris pH 8.0, 150 mM NaCl, 10% glycerol, and 10 mM β‐mercaptoethanol). The protein was concentrated to 2 mL and loaded onto a Superdex 75 size exclusion column with buffer 6 (20 mM Tris pH 8.0, 150 mM NaCl, 10% glycerol, and 10 mM DTT). The eluted protein was collected, pooled, and concentrated to a final concentration of 10 mg/mL.

### Binding assay

The binding affinity was evaluated using a Microscale Thermophoresis system, Monolith NT.115 (NanoTemper, Watertown, MA, USA). The experiments involved a 1:1 mixture of recombinant PPAR𝛼 and DhT (dissolved in DMSO) in standard label grade capillaries. A 250 nM protein concentration was titrated against a serial dilution of DhT (ranging from 0 to 500 μM) to generate 16 samples (using a two‐fold dilution) with a total volume of 10 µL. Measurements were conducted at 25 °C with 20% MST power. The thermophoresis data were analyzed using the Kd model assuming 1:1 binding, utilizing NanoTemper's MO Affinity V2.3 software.

### Luciferase reporter assay

The promoter regions of PPAR𝛼 (1500 bp from ATG) and catalase (2000 bp from ATG) were synthesized by TWSIT Bioscience (South San Francisco, CA, USA) and subsequently ligated into the pGL3 Basic Vector (Promega, Madison, WI, USA). The wild type full‐length PPAR𝛼 sequence (GenBank accession number NM_005036.6) was synthesized by Integrated DNA Technologies (San Diego, CA, USA), and Mutated full length PPAR𝛼 was obtained PCR‐aided site directed mutation (T283A). Both wild type and mutated PPAR𝛼 sequences were inserted into the pcDNA™3.1(+) vector (V79020, Thermo Fisher Scientific). Transfection of each of the plasmids, along with a control pEGFP‐C1 plasmid (V012024, NovoPro Bioscience, Shanghai, China), into HepG2 cells was accomplished using Lipofectamine 2000 (11668027, Thermo Fisher Scientific). After overnight transfection, the medium was replaced with fresh medium containing CHO (10 μM) and/or DhT (1.0 µg/mL). Sample preparation and signal detection were conducted following the methodology outlined in a previous publication.^[49c]^

### Determination of CHO and major oxysterols using Gas Chromatography‐Mass Spectrometry

Liver tissues collected from ND and HCD mice were homogenized and 5α‐cholestane (internal standard, C8003, Sigma‐Aldrich) was added. The mixture was then extracted three times with isopropanol. The combined extraction was subject to N, O‐bis‐trimethylsilyl‐trifluoroacetamide with 1% trimethylchlorosilane (BSTFA‐TMCS, T6381, Sigma‐Aldrich) derivatization (Valverde‐Som et al., 2018). GC‐MS analysis was performed using an Agilent GC‐MS system (7890B, Agilent Technologies, Santa Clara, CA, USA) following the procedure outlined in a previous publication (ref. 62). Quantification was carried out with reference to standard chemicals, including Cholesterol (sc‐202539A, Santa Cruz); 27‐Hydroxycholesterol (sc‐358756A, Santa Cruz), 25‐Hydroxycholesterol (sc‐214091, Santa Cruz), 24‐Hydroxycholesterol (sc‐489589, Santa Cruz), 7‐Ketocholesterol (sc‐210630, Santa Cruz), and 7β‐Hydroxy Cholesterol (sc‐210655, Santa Cruz).

### Data analysis

Data from a minimum of three independent experiments were analyzed using one‐way ANOVA and Student's t‐test with GraphPad Prism Version 10.1.0 (264) (GraphPad Software, Boston, MA, USA). The data were presented as means ± SD. P values less than 0.05 were considered statistically significant.

## Conflict of Interest

The authors declare no conflict of interest.

## Author Contributions

Jia‐Wen Shou: conceptualization, investigation, methodology, validation, formal analysis, data curation and writing – original draft, and writing – review & editing. Juncai Ma and Byung‐Ho Kang: transmission electron microscopy analysis. Xiao‐Xiao Li: Biochemical parameter analysis. Shu‐Cheng Chen: conceptualization and discussion. Pang‐Chui Shaw: conceptualization, supervision, writing – original draft, and writing – review & editing.

## Supporting information

Supporting Information

## Data Availability

The data that support the findings of this study are available on request from the corresponding author. The data are not publicly available due to privacy or ethical restrictions
