# Supplementary material for: Free Cholesterol‐Induced Liver Injury in Non‐Alcoholic Fatty Liver Disease: Mechanisms and a Therapeutic Intervention Using Dihydrotanshinone I
Source: Adv Sci (Weinh). 2024 Nov 19;12(2):2406191. doi: 10.1002/advs.202406191 (PMC11727260; doi:10.1002/advs.202406191)
Supplement: Supplementary file 1 — Supporting Information [file ADVS-12-2406191-s001.pdf]

ADVANCED SCIENCE
Open Access

Supporting Information

for *Adv. Sci.*, DOI 10.1002/advs.202406191

Free Cholesterol-Induced Liver Injury in Non-Alcoholic Fatty Liver Disease: Mechanisms and a Therapeutic Intervention Using Dihydrotanshinone I

*Jia-Wen Shou, Juncai Ma, Xuchu Wang, Xiao-Xiao Li, Shu-Cheng Chen, Byung-Ho Kang and Pang-Chui Shaw**

Free Cholesterol-Induced Liver Injury in Non-Alcoholic Fatty Liver Disease: Mechanisms and a Therapeutic Intervention Using Dihydrotanshinone I

Jia-Wen Shou[1], Juncai Ma[2], Xuchu Wang[3], Xiao-Xiao Li[1,4], Shu-Cheng Chen[5], Byung-Ho Kang[2], Pang-Chui Shaw[1,6,7,*]

1 Li Dak Sum Yip Yio Chin R&D Centre for Chinese Medicine, The Chinese University of Hong Kong, Hong Kong, 852852, China.
2 Centre for Cell and Developmental Biology, State Key Laboratory for Agrobiotechnology, School of Life Sciences, The Chinese University of Hong Kong, Hong Kong, 852852, China
3 Department of Laboratory Medicine, the Second Affiliated Hospital of Zhejiang University, Hangzhou, 310000, China.
4 Research Center for Chinese Medicine Innovation, The Hong Kong Polytechnic University, Hong Kong, 852852, China.
.
5 School of Nursing, The Hong Kong Polytechnic University, Hong Kong, 852852, China.
6 School of Life Sciences, The Chinese University of Hong Kong, Hong Kong, 852852, China.
7 State Key Laboratory of Research on Bioactivities and Clinical Applications of Medicinal Plants and Institute of Chinese Medicine, The Chinese University of Hong Kong, Hong Kong, 852852, China.
*Corresponding author. Address: Room 180, Science Centre South Block, The Chinese University of Hong Kong, Shatin, New Territories, Hong Kong, 852852, China; Tel.: +852 3943 1363, fax: +852 2603 7246. E-mail address: pcshaw@cuhk.edu.hk.

Table S1

NCBI Reference Sequence: NM_001113418.1		
shRNA-1	GGAAAGTCCCTTATCTGAA	
shRNA-2	GCAATTCGCTTTGGAAGAA	
shRNA-3	GCCTGGCCTTCTAAACATA	
AAV titer	ITR-F	GGAACCCCTAGTGATGGAGTT
	ITR-R	CGGCCTCAGTGAGCGA

Table S2 qPCR primer

Species	Sequence		Accession number
Bacteria	16S rRNA	F: ATGGYTGTCGTCAGCTCGTG	
		R: GGGTTGCGCTCGTTGC	
Mouse	*Fatp1*	F: GCACAGCAGGTACTACCGCA	NM_001357182.2
		R: GGCGGCACGCATGCTG	
	Cap1α	F: AGATCAATCGGACCCTAGACAC	XM_036161417.1
		R: CAGCGAGTAGCGCATAGTCA	
	Acox1	F: CAAGACCCAAGAGTTCATT	NM_015729.4
		R: TTCAGGTAGCCATTATCCA	
Human	*Catalase*	F: GCTCTTCTGGACAAGTACAATGCTG	NM_001752.4
		R: TTACACGGATGAACGCTAAGCTTC	
	SOD1	F: ACTGGTGGTCCATGAAAAAGC	NM_000454.5
		R: AACGACTTCCAGCGTTTCCT	
	SOD2	F: CTGATTTGGACAAGCAGCAA	NM_001322819.2
		R: CTGGACAAACCTCAGCCCTA	
	TRX1	F: ACGCTGCAGGTGATAAAC	NM_003329.4
		R: CTGACAGTCATCCACATCTAC	
	HO-1	F: CTCAAACCTCCAAAAGCC	NM_002133.3
		R: TCAAAAACCACCCCAACCC	
	GPX4	F: GAGGCAAGACCGAAGTAAACTAC	NM_001367832.1
		R: CCGAACTGGTTACACGGGAA	
Mouse	*β-Actin*	F: TGTCCCTGTATGCCT	NM_007393.5
		R: TCACGCACGATTTCCCTC	
Human	*β-ACTIN*	F: TCCCTGGAGAAGAGCTACGA	NM_001101.5
		R: AGCACTGTGTTGGCGTACAG	

Figure legends

Figure S1. (A-B) Age (A) and sex (B) information of all the participants. (C) Plasma levels of triglycerides, TC, HDL-c and LDL-c in all the participants. (D-I) Mice weight (D), liver index (E), liver morphology (F), NAFLD score and fibrosis area (G), hepatic TG content (H) and levels of hepatic inflammatory markers (I) with ND or HCD administration. (J-K) Plasma level of c-CASP3 (J) and catalase (K) in NAFLD patient without and with treatment.

Figure S2. (A) Quantification of oxysterols and CHO in mice liver using GC. (B) Immunoblotting of cholesterol esterase in HepG2 cell challenged with CHO. (C) Cell viability with CHO or cholesterol esters treatment. (D-E) Flippin staining of FC and immunohistology staining of TUNEL (E) in mouse liver sections, scale bar, 100μm. (F-G) Cell viability and hepatic c-CASP3 level with CHO and/or autophagic inhibitors treatment in HepG2 cells (F) and primary hepatocytes (G).

Figure S3. (A-B) Immunoblotting and quantification of CTSB and CTSD in HepG2 cells (A) and primary hepatocytes (B) without and with CHO treatment. (C) Quantification of mature CTSB and CTSD immunoblots in HepG2 cells and primary hepatocytes without and with CHO treatment. (D) Quantification of hepatic P62, ratio of LC3BII to I, c-CASP3, mature CTSB and CTSD immunoblots in in mice fed with ND or HCD.

Figure S4. (A) Differential expressed genes from HCD mice versus ND mice. (B-D) GSEA analysis of autophagy pathway (B), apoptosis pathway (C) and inflammatory response (D) in HCD and ND mice. (E) Quantification of P62, LC3B, c-CASP3, CTSB and CTSD in HepG2 cells with CHO, NAC or mito-TEMPO treatment.

Figure S5. (A-B) Alpha diversity (A, ace; B, chao) of gut microbiota in mice feces. (C) Principal component analysis of gut microbiota in mice feces. (D) Hierarchy of gut microbiota in mice feces at the phylum level. (E) Heatmap of top 50 gut microbial families in mice feces. (F-G) Gut microbiota health index in mice feces.

Figure S6. (A) Experimental grouping information in Abs-treated mice. (B) qPCR analysis of fecal 16S rDNA level. (C-D) Mouse weight (C) and liver index (D) on day 56. (E) H&E, oil red O and Sirius red staining of mouse liver sections. (F-G) NAFLD score (F) and fibrosis area (G) in mouse liver. (H-I) Liver function indices, ALT (H) and total bilirubin (I). (J, L) Hepatic levels of TG (J), TC, FC and CE (L). (K, M) Hepatic ROS (K) and antioxidases (M) level. (N) Hepatic inflammatory markers level.

Figure S7. (A) Fecal TC content. (B) Cell viability of HepG2 cells and primary hepatocytes with CHO and DhT treatment. (C-D) Immunoblotting (C) and quantification (D) of hepatic P62, LC3B, c-CASP3, CTSB and CTSD in HepG2 cells and primary hepatocytes with CHO and DhT treatment. (E) TUNEL staining in mouse liver sections, scale bar: 50 μm.

Figure S8. (A) PPARs expression in HepG2 and primary hepatocytes with CHO and DhT treatment. (B) PPARs levels in siRNA-transfected cells (HepG2 and primary hepatocytes) with CHO and DhT treatment. (C) Expressions of catalase, P62, LC3B, c-CASP3, mature CTSB and mature CTSD in siRNA-transfected cells (HepG2 and primary hepatocytes) with CHO and DhT treatment. (D) Hepatic mRNA levels of Fatp1, Cpt1α and Acox1 in mice with ND, HCD and DhT treatment. (E) mRNA levels of antioxidant genes in siRNA-transfected HepG2 cells with CHO and DhT treatment. (F) Quantifications of PPARα, catalase, P62, LC3B, c-CASP3, mature CTSB and mature CTSD in AAV mouse liver.

Figure S9. (A-F) Recombinant protein expression, purification and concertation of PPARα LBD wild type (A-C) and mutant (D-F). (A-B) L: protein ladder; 1: lysate; 2: flow through; 3: wash1; 4: wash2; 5: wash3; 6: wash4; 7: elution with 25 mM imidazole; 8: elution with 50 mM imidazole; 9: elution with 100 mM imidazole; 10: elution with 200 mM imidazole; 11:

elution with 250 mM imidazole; 12: elution with 300 mM imidazole; 13: elution with 500 mM imidazole. (B, E) L: protein ladder; 1: concentrated protein before gel filtration; 2: flowthrough during concentration; 3: fraction 19; 4: fraction 20; 5: fraction 21; 6: fraction 22; 7: fraction 23; 8: fraction 24; 9: fraction 25; 10: fraction 26; 11: fraction 27; 12: fraction 28; 13: fraction 29. (C, F) Concentrated protein after gel filtration, L: protein ladder; 1: 2X dilution; 2: 5X dilution; 3: 10X dilution.

Figure S10. PPARα shRNA efficiency. PPARα expression in murine cell lines (C17.2 and 3T3L1 cells) with three pieces of shRNA treatment (left) and different doses of shRNA-1 treatment (right).

Figure S1

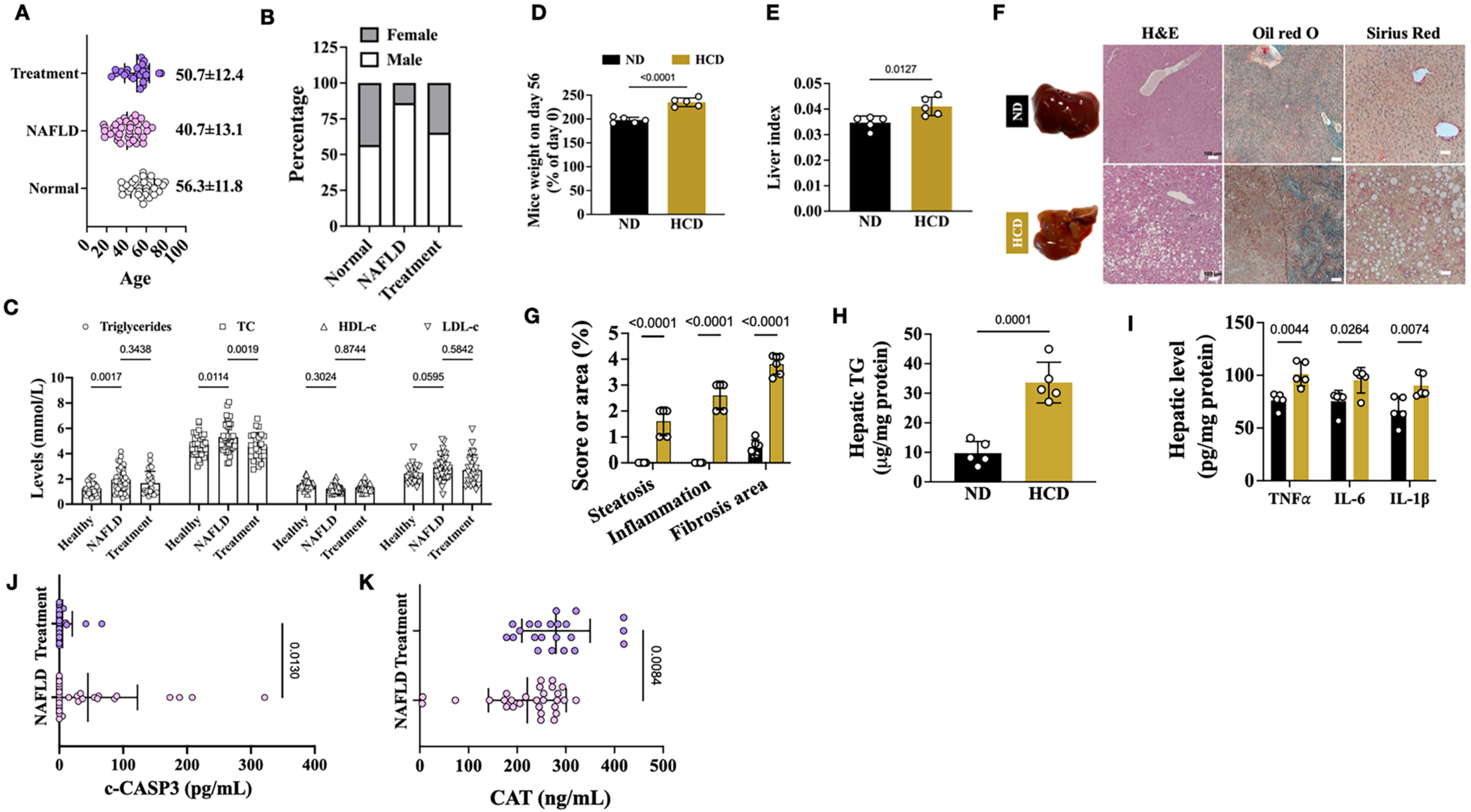

A
Treatment
NAFLD
Normal
50.7±12.4
40.7±13.1
56.3±11.8
Age
B
Female
Male
Percentage
Normal
NAFLD
Treatment
C
Triglycerides
TC
HDL-c
LDL-c
Levels (mmol/L)
0.0017
0.3438
0.0114
0.0019
0.3024
0.8744
0.0595
0.5842
Healthy
NAFLD
Treatment
D
ND
HCD
Mice weight on day 56 (% of day 0)
<0.0001
E
Liver index
0.0127
F
H&E
Oil red O
Sirius Red
ND
HCD
G
Score or area (%)
<0.0001
Steatosis
Inflammation
Fibrosis area
H
Hepatic TG (μg/mg protein)
0.0001
I
Hepatic level (pg/mg protein)
0.0044
0.0264
0.0074
TNFα
IL-6
IL-1β
J
NAFLD
Treatment
0.0130
c-CASP3 (pg/mL)
K
CAT (ng/mL)
0.0084

Figure S2

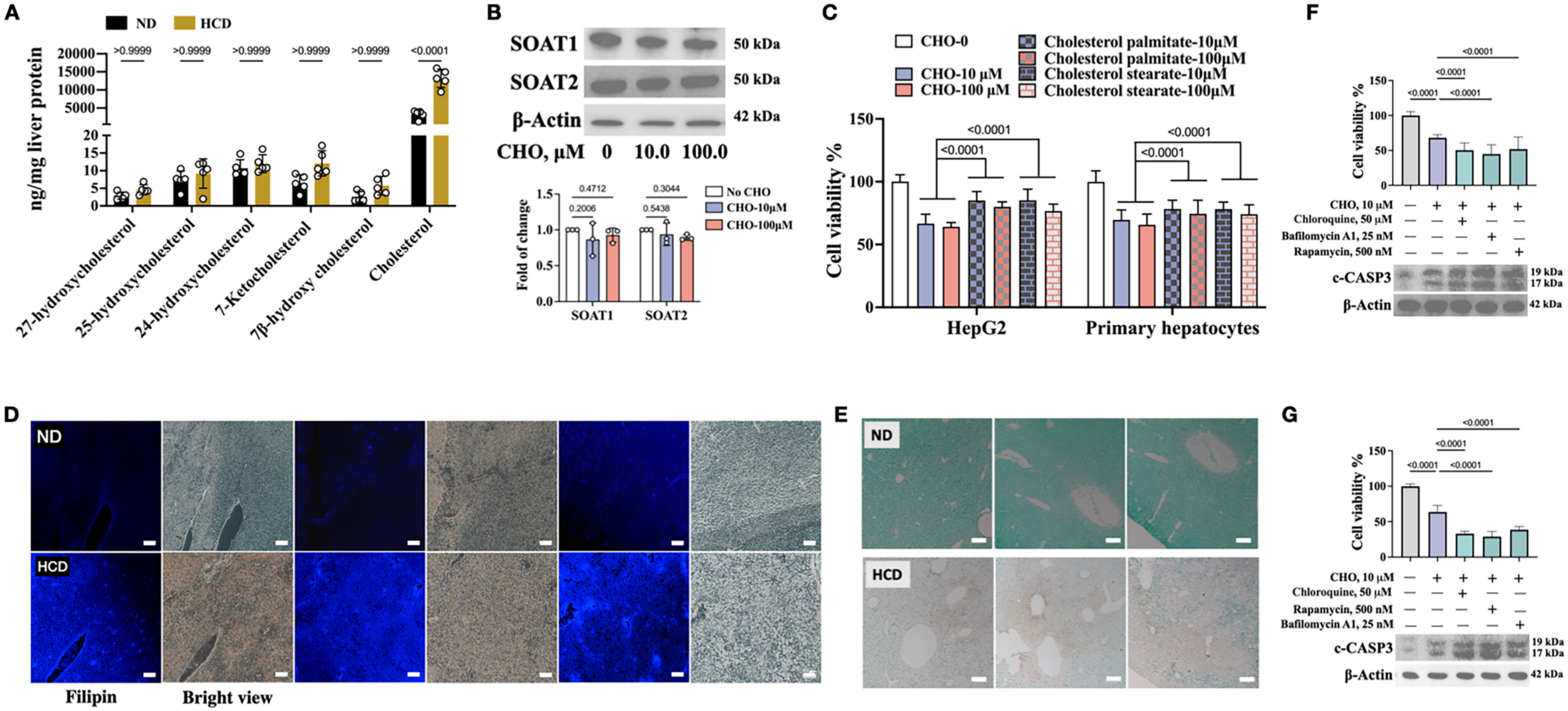

A
ND
HCD
ng/mg liver protein
>0.9999
<0.0001
27-hydroxycholesterol
25-hydroxycholesterol
24-hydroxycholesterol
7-Ketocholesterol
7β-hydroxy cholesterol
Cholesterol
B
SOAT1
SOAT2
β-Actin
50 kDa
42 kDa
CHO, μM 0 10.0 100.0
Fold of change
No CHO
CHO-10μM
CHO-100μM
C
CHO-0
CHO-10 μM
CHO-100 μM
Cholesterol palmitate-10μM
Cholesterol palmitate-100μM
Cholesterol stearate-10μM
Cholesterol stearate-100μM
Cell viability %
HepG2
Primary hepatocytes
D
ND
HCD
Filipin
Bright view
E
F
CHO, 10 μM
Chloroquine, 50 μM
Bafilomycin A1, 25 nM
Rapamycin, 500 nM
c-CASP3
19 kDa
17 kDa
G

Figure S3

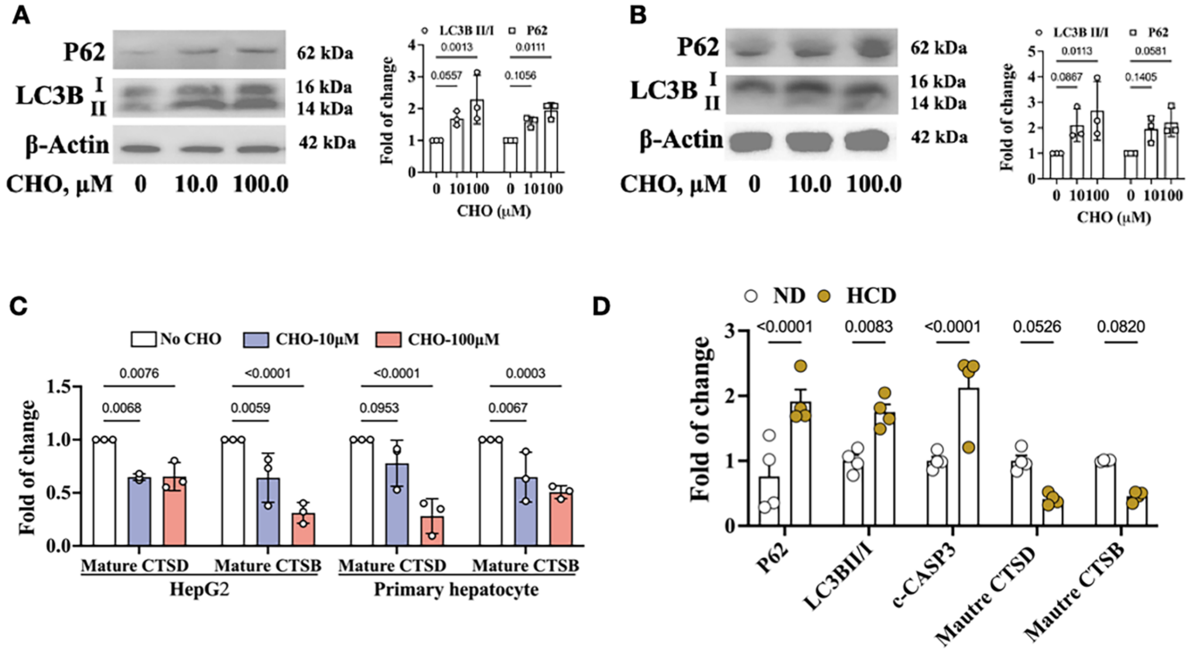

A
P62
62 kDa
LC3B I II
16 kDa
14 kDa
β-Actin
42 kDa
CHO, µM 0 10.0 100.0
LC3B II/I
P62
Fold of change
CHO (µM)
B
P62
62 kDa
LC3B I II
16 kDa
14 kDa
β-Actin
42 kDa
CHO, µM 0 10.0 100.0
LC3B II/I
P62
Fold of change
CHO (µM)
C
No CHO
CHO-10µM
CHO-100µM
Fold of change
Mature CTSD
Mature CTSB
HepG2
Primary hepatocyte
D
ND
HCD
Fold of change
P62
LC3BII/I
c-CASP3
Mautre CTSD
Mautre CTSB

Figure S4

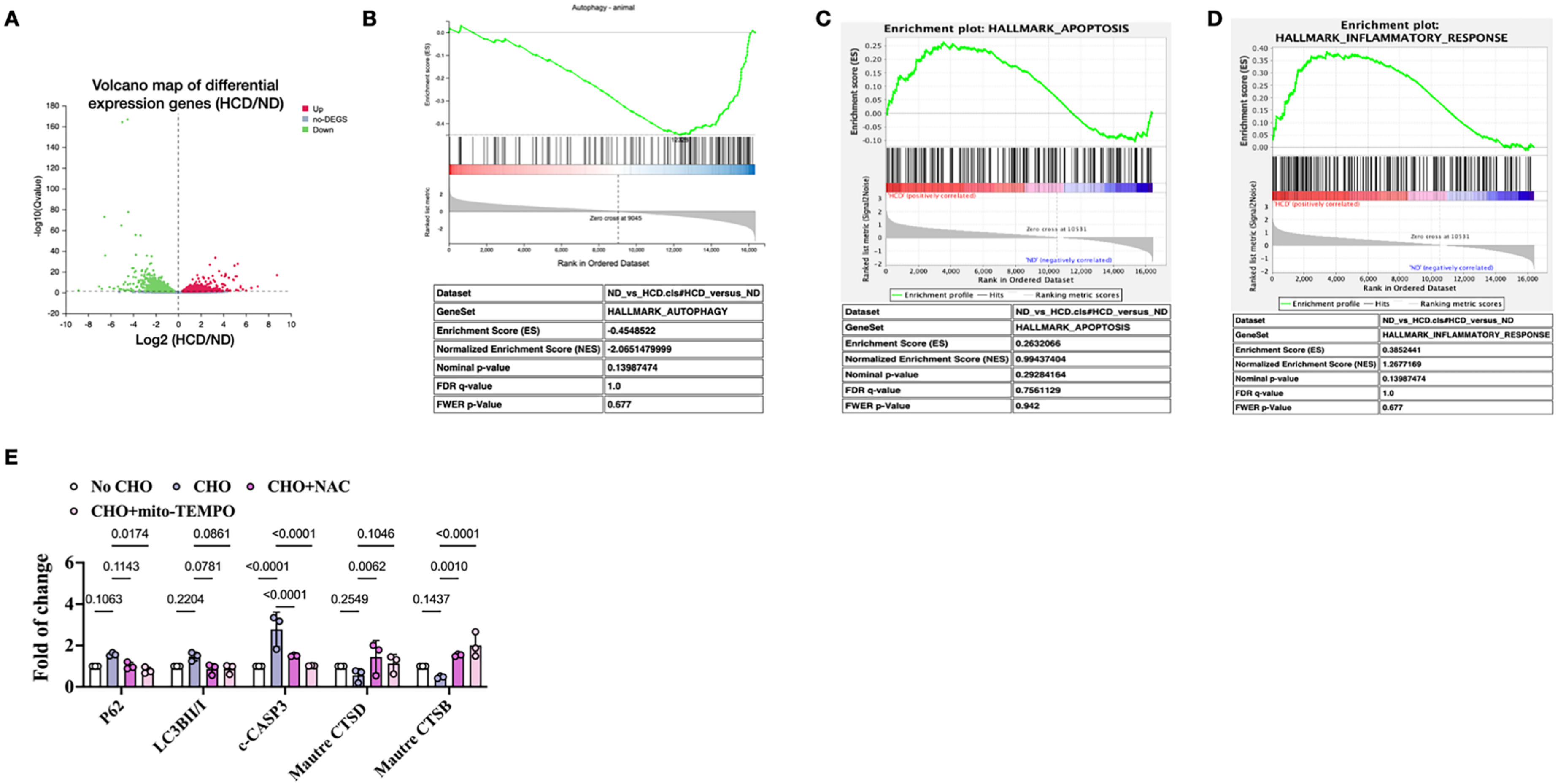
A
Volcano map of differential expression genes (HCD/ND)
Up
no-DEGS
Down
-log10(Qvalue)
Log2 (HCD/ND)
B
Autophagy - animal
Enrichment score (ES)
Ranked list metric
Zero cross at 9045
Rank in Ordered Dataset
Dataset
ND_vs_HCD.cls#HCD_versus_ND
GeneSet
HALLMARK_AUTOPHAGY
Enrichment Score (ES)
-0.4548522
Normalized Enrichment Score (NES)
-2.0651479999
Nominal p-value
0.13987474
FDR q-value
1.0
FWER p-Value
0.677
C
Enrichment plot: HALLMARK_APOPTOSIS
Enrichment score (ES)
Ranked list metric (Signal2Noise)
'HCD' (positively correlated)
Zero cross at 10531
'ND' (negatively correlated)
Rank in Ordered Dataset
Enrichment profile
Hits
Ranking metric scores
Dataset
ND_vs_HCD.cls#HCD_versus_ND
GeneSet
HALLMARK_APOPTOSIS
Enrichment Score (ES)
0.2632066
Normalized Enrichment Score (NES)
0.99437404
Nominal p-value
0.29284164
FDR q-value
0.7561129
FWER p-Value
0.942
D
Enrichment plot:
HALLMARK_INFLAMMATORY_RESPONSE
Enrichment score (ES)
Ranked list metric (Signal2Noise)
'HCD' (positively correlated)
Zero cross at 10531
'ND' (negatively correlated)
Rank in Ordered Dataset
Enrichment profile
Hits
Ranking metric scores
Dataset
ND_vs_HCD.cls#HCD_versus_ND
GeneSet
HALLMARK_INFLAMMATORY_RESPONSE
Enrichment Score (ES)
0.3852441
Normalized Enrichment Score (NES)
1.2677169
Nominal p-value
0.13987474
FDR q-value
1.0
FWER p-Value
0.677
E
No CHO
CHO
CHO+NAC
CHO+mito-TEMPO
Fold of change
0.0174
0.1143
0.1063
0.0861
0.0781
0.2204
<0.0001
<0.0001
<0.0001
0.1046
0.0062
0.2549
<0.0001
0.0010
0.1437
P62
LC3BII/I
c-CASP3
Mautre CTSD
Mautre CTSB

Figure S5

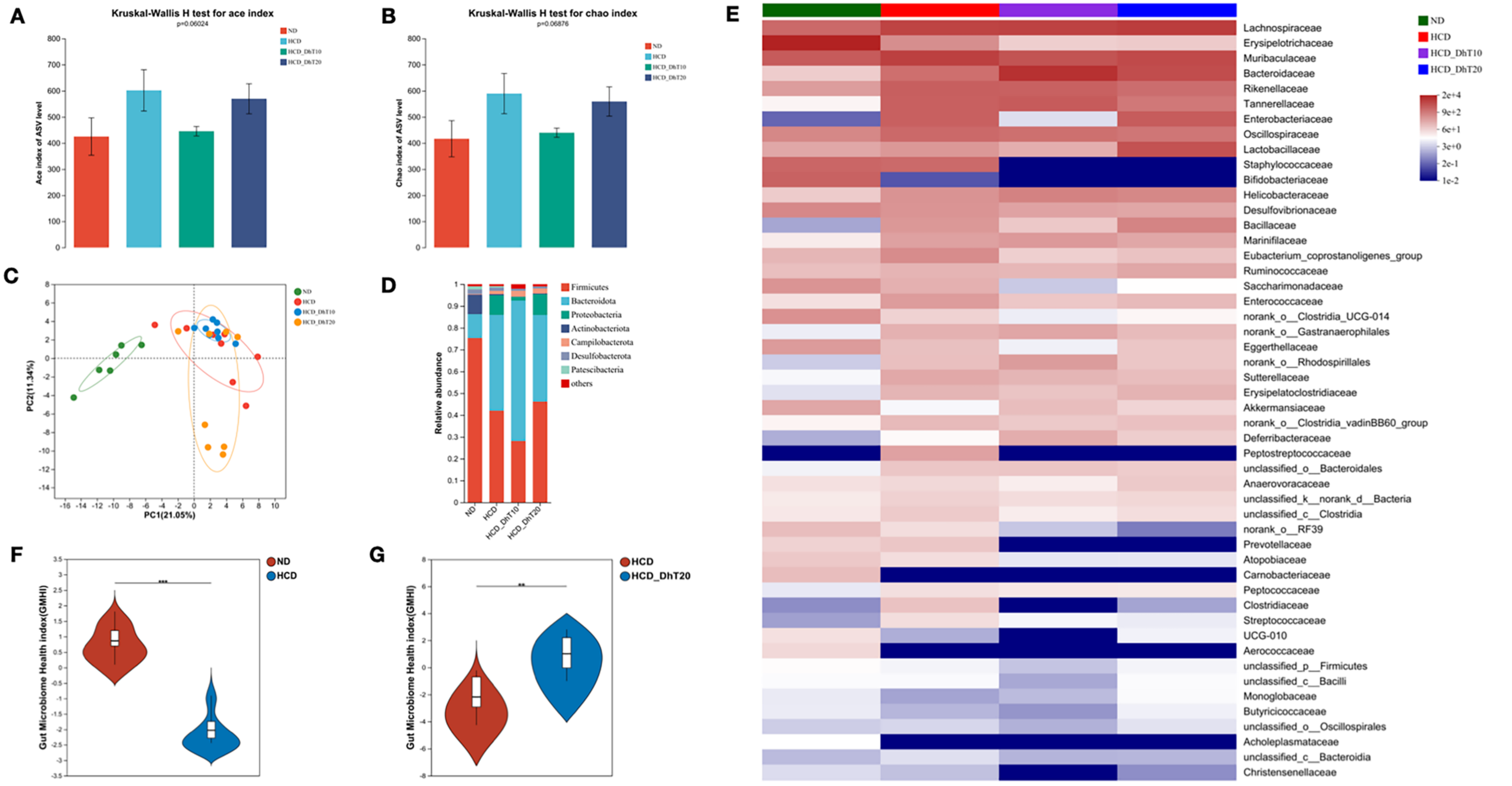

A
Kruskal-Wallis H test for ace index
p=0.06024
ND
HCD
HCD_DhT10
HCD_DhT20
Ace index of ASV level
B
Kruskal-Wallis H test for chao index
p=0.06876
Chao index of ASV level
C
PC1(21.05%)
PC2(11.34%)
D
Relative abundance
Firmicutes
Bacteroidota
Proteobacteria
Actinobacteriota
Campilobacterota
Desulfobacterota
Patescibacteria
others
E
Lachnospiraceae
Erysipelotrichaceae
Muribaculaceae
Bacteroidaceae
Rikenellaceae
Tannerellaceae
Enterobacteriaceae
Oscillospiraceae
Lactobacillaceae
Staphylococcaceae
Bifidobacteriaceae
Helicobacteraceae
Desulfovibrionaceae
Bacillaceae
Marinifilaceae
Eubacterium_coprostanoligenes_group
Ruminococcaceae
Saccharimonadaceae
Enterococcaceae
norank_o__Clostridia_UCG-014
norank_o__Gastranaerophilales
Eggerthellaceae
norank_o__Rhodospirillales
Sutterellaceae
Erysipelatoclostridiaceae
Akkermansiaceae
norank_o__Clostridia_vadinBB60_group
Deferribacteraceae
Peptostreptococcaceae
unclassified_o__Bacteroidales
Anaerovoracaceae
unclassified_k__norank_d__Bacteria
unclassified_c__Clostridia
norank_o__RF39
Prevotellaceae
Atopobiaceae
Carnobacteriaceae
Peptococcaceae
Clostridiaceae
Streptococcaceae
UCG-010
Aerococcaceae
unclassified_p__Firmicutes
unclassified_c__Bacilli
Monoglobaceae
Butyricicoccaceae
unclassified_o__Oscillospirales
Acholeplasmataceae
unclassified_c__Bacteroidia
Christensenellaceae
2e+4
9e+2
6e+1
3e+0
2e-1
1e-2
F
Gut Microbiome Health index(GMHI)
***
G
**

Figure S6

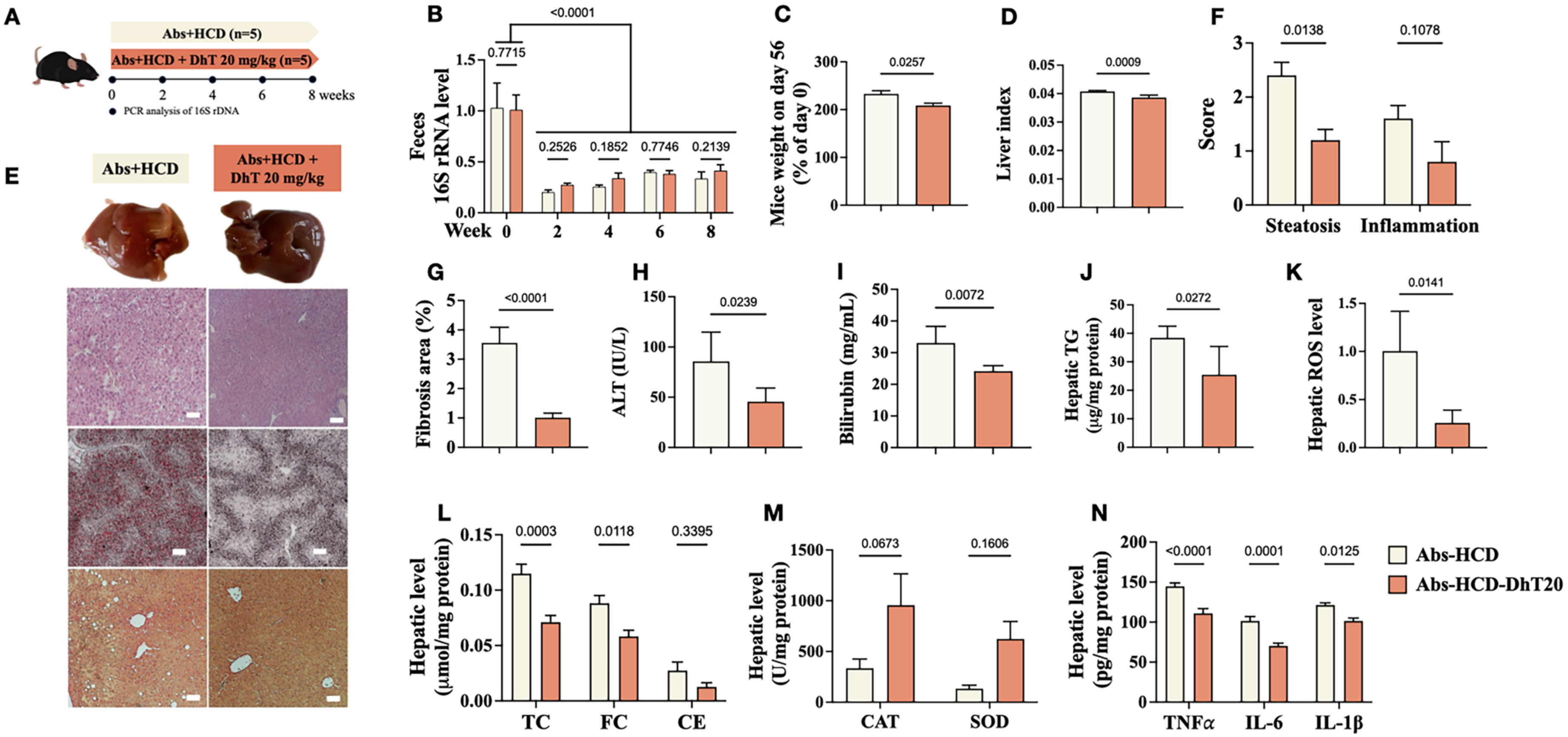
A
Abs+HCD (n=5)
Abs+HCD + DhT 20 mg/kg (n=5)
0 2 4 6 8 weeks
PCR analysis of 16S rDNA
B
<0.0001
0.7715
0.2526 0.1852 0.7746 0.2139
Feces 16S rRNA level
Week 0 2 4 6 8
C
Mice weight on day 56 (% of day 0)
0.0257
D
Liver index
0.0009
F
Score
0.0138 0.1078
Steatosis Inflammation
E
Abs+HCD
Abs+HCD + DhT 20 mg/kg
G
Fibrosis area (%)
<0.0001
H
ALT (IU/L)
0.0239
I
Bilirubin (mg/mL)
0.0072
J
Hepatic TG (μg/mg protein)
0.0272
K
Hepatic ROS level
0.0141
L
Hepatic level (μmol/mg protein)
0.0003 0.0118 0.3395
TC FC CE
M
Hepatic level (U/mg protein)
0.0673 0.1606
CAT SOD
N
Hepatic level (pg/mg protein)
<0.0001 0.0001 0.0125
TNFα IL-6 IL-1β
Abs-HCD
Abs-HCD-DhT20

Figure S7

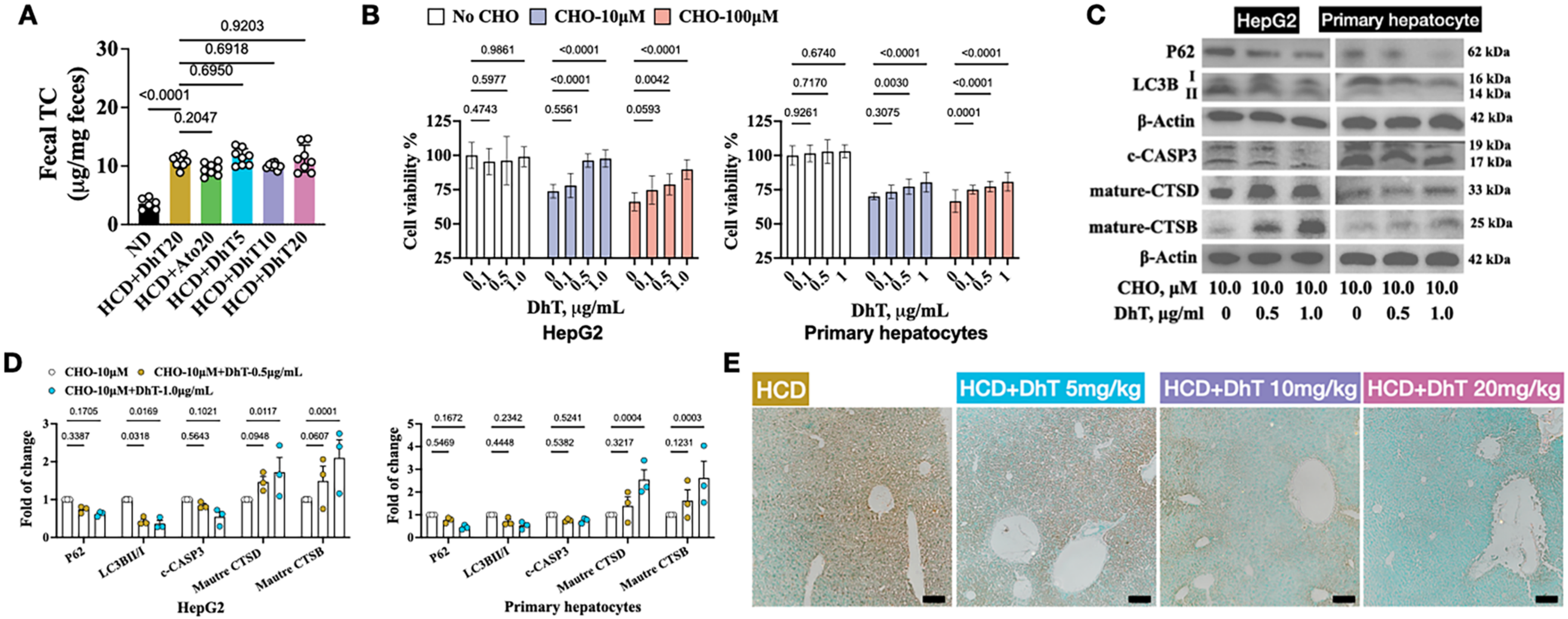
A
B
C
D
E
Fecal TC (μg/mg feces)
No CHO
CHO-10μM
CHO-100μM
Cell viability %
DhT, μg/mL
HepG2
Primary hepatocytes
Primary hepatocyte
P62
LC3B
β-Actin
c-CASP3
mature-CTSD
mature-CTSB
CHO, μM
DhT, μg/ml
CHO-10μM
CHO-10μM+DhT-0.5μg/mL
CHO-10μM+DhT-1.0μg/mL
Fold of change
HCD
HCD+DhT 5mg/kg
HCD+DhT 10mg/kg
HCD+DhT 20mg/kg

Figure S8

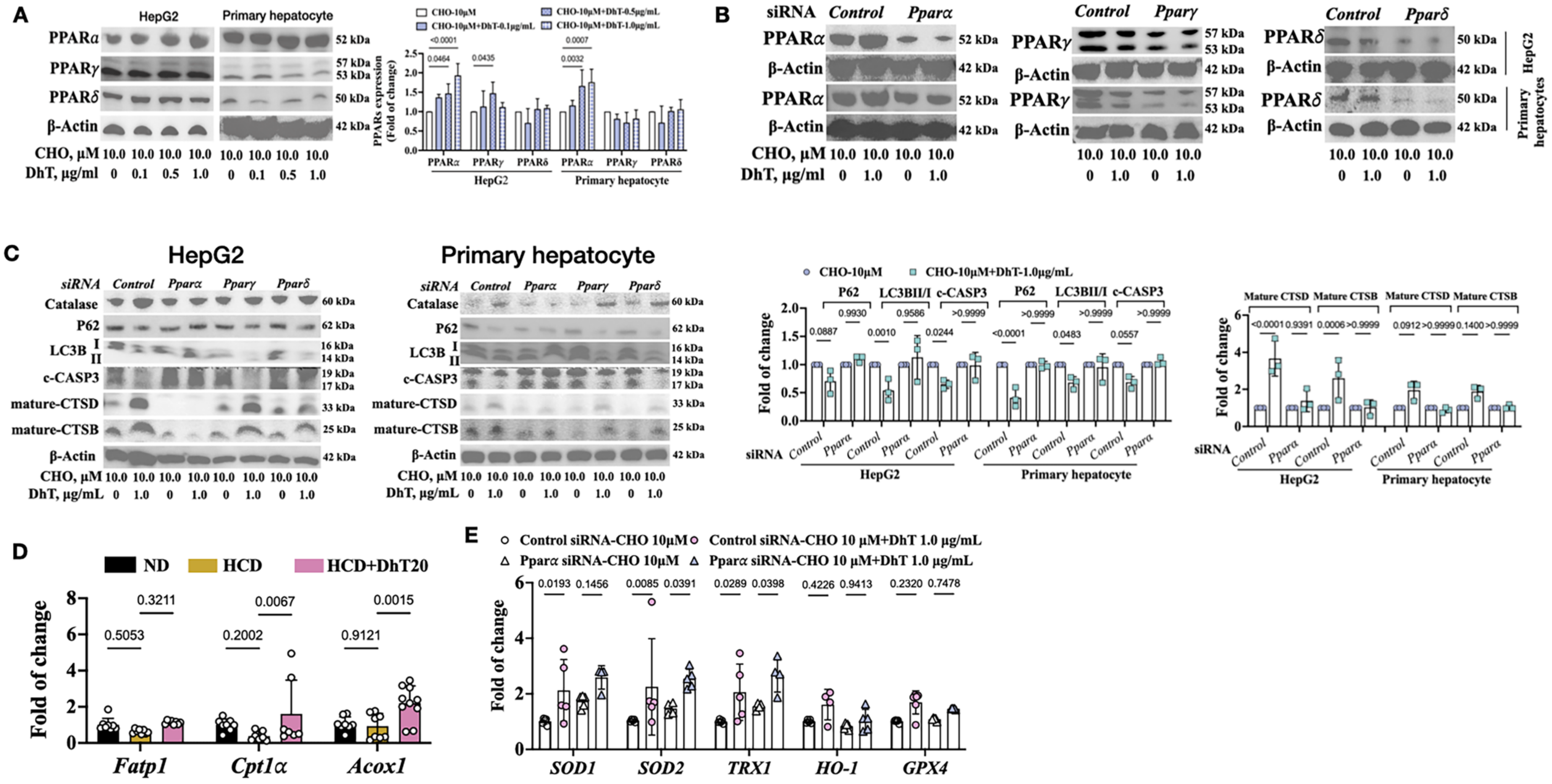
A
HepG2
Primary hepatocyte
PPARα
PPARγ
PPARδ
β-Actin
CHO, μM
DhT, μg/ml
CHO-10μM
CHO-10μM+DhT-0.1μg/mL
CHO-10μM+DhT-0.5μg/mL
CHO-10μM+DhT-1.0μg/mL
PPARs expression (Fold of change)
B
siRNA Control Pparα
Control Pparγ
Control Pparδ
HepG2
Primary hepatocytes
C
HepG2
Primary hepatocyte
Catalase
P62
LC3B
c-CASP3
mature-CTSD
mature-CTSB
CHO-10μM
CHO-10μM+DhT-1.0μg/mL
Fold of change
Mature CTSD
Mature CTSB
D
ND
HCD
HCD+DhT20
Fold of change
Fatp1
Cpt1α
Acox1
E
Control siRNA-CHO 10μM
Control siRNA-CHO 10 μM+DhT 1.0 μg/mL
Pparα siRNA-CHO 10μM
Pparα siRNA-CHO 10 μM+DhT 1.0 μg/mL
Fold of change
SOD1
SOD2
TRX1
HO-1
GPX4

Figure S9

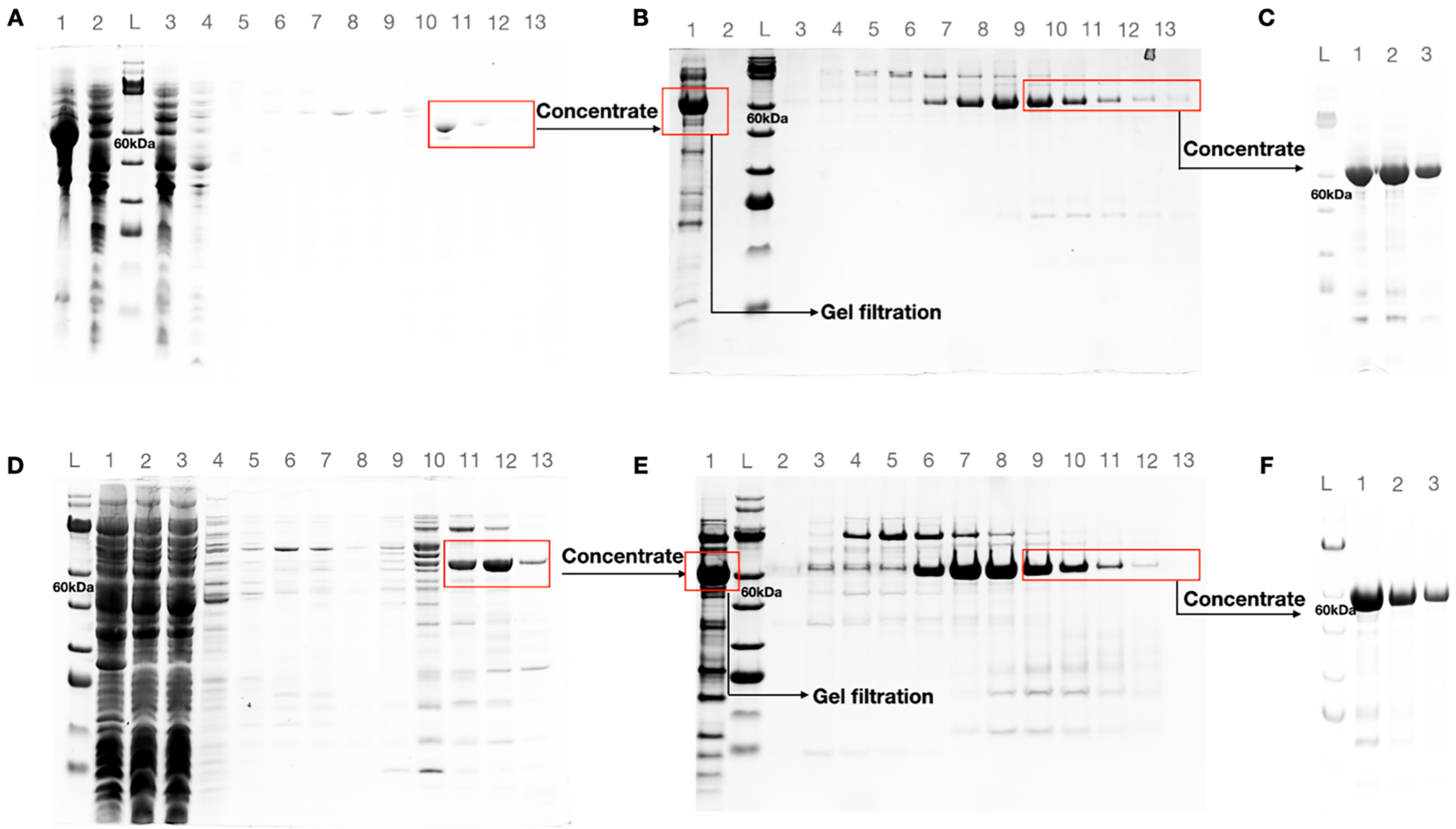

A
1 2 L 3 4 5 6 7 8 9 10 11 12 13
60kDa
Concentrate
B
1 2 L 3 4 5 6 7 8 9 10 11 12 13
60kDa
Concentrate
Gel filtration
C
L 1 2 3
60kDa
D
L 1 2 3 4 5 6 7 8 9 10 11 12 13
60kDa
Concentrate
E
1 L 2 3 4 5 6 7 8 9 10 11 12 13
60kDa
Concentrate
Gel filtration
F
L 1 2 3
60kDa

Figure S10

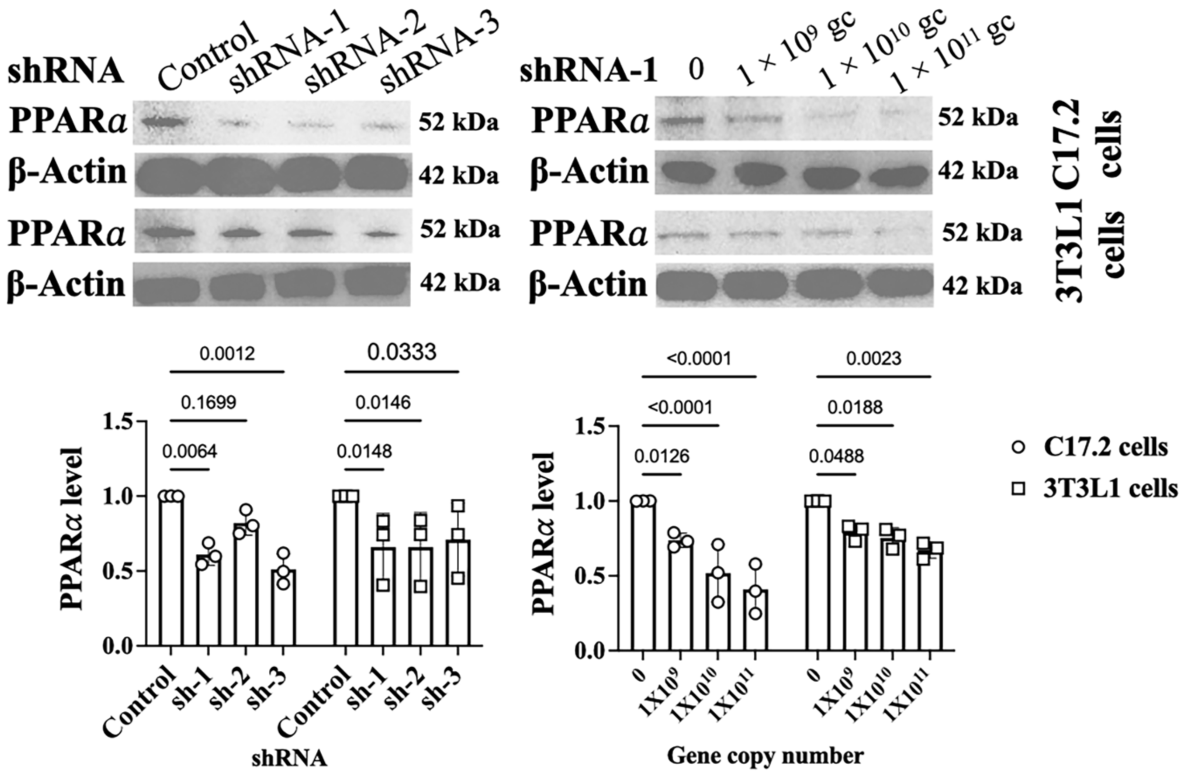
shRNA
Control
shRNA-1
shRNA-2
shRNA-3
PPARα
β-Actin
52 kDa
42 kDa
shRNA-1
0
1 × 10^9 gc
1 × 10^10 gc
1 × 10^11 gc
C17.2 cells
3T3L1 cells
0.0012
0.1699
0.0064
0.0333
0.0146
0.0148
PPARα level
Control
sh-1
sh-2
sh-3
shRNA
<0.0001
<0.0001
0.0126
0.0023
0.0188
0.0488
1X10^9
1X10^10
1X10^11
Gene copy number
C17.2 cells
3T3L1 cells
